# Supplementary material for: idpr: A package for profiling and analyzing Intrinsically Disordered Proteins in R
Source: PLoS One. 2022 Apr 18;17(4):e0266929. doi: 10.1371/journal.pone.0266929 (PMC9015136; doi:10.1371/journal.pone.0266929)
Supplement: S1 File — (DOCX) [file pone.0266929.s001.docx]

**Supplementary Information for *idpr*: A package for profiling and analyzing Intrinsically Disordered Proteins in R.**

William M. McFadden^1,#^ and Judith L. Yanowitz^1,2,*^

^1^Magee-Womens Research Institute, University of Pittsburgh Medical Center, Pittsburgh, PA, USA;

^2^University of Pittsburgh School of Medicine, Pittsburgh, PA, USA;

^#^Current Address: Laboratory of Biochemical Pharmacology, Department of Pediatrics, Emory University School of Medicine, Atlanta, GA, USA

^*^Corresponding Author; Email: yanowitzjl@mwri.magee.edu

**This PDF file includes:**

List of abbreviations

Figures S1 and S2

Captions for S1 and S2

References for this document

**List of Abbreviations**

αSyn α-Synuclein

CTD C-terminal Domain

DBD DNA Binding Domain

GCNA Germ Cell Nuclear Antigen

IDP Intrinsically Disordered Protein

IDR Intrinsically Disordered Region

NTD N-terminal Domain

**
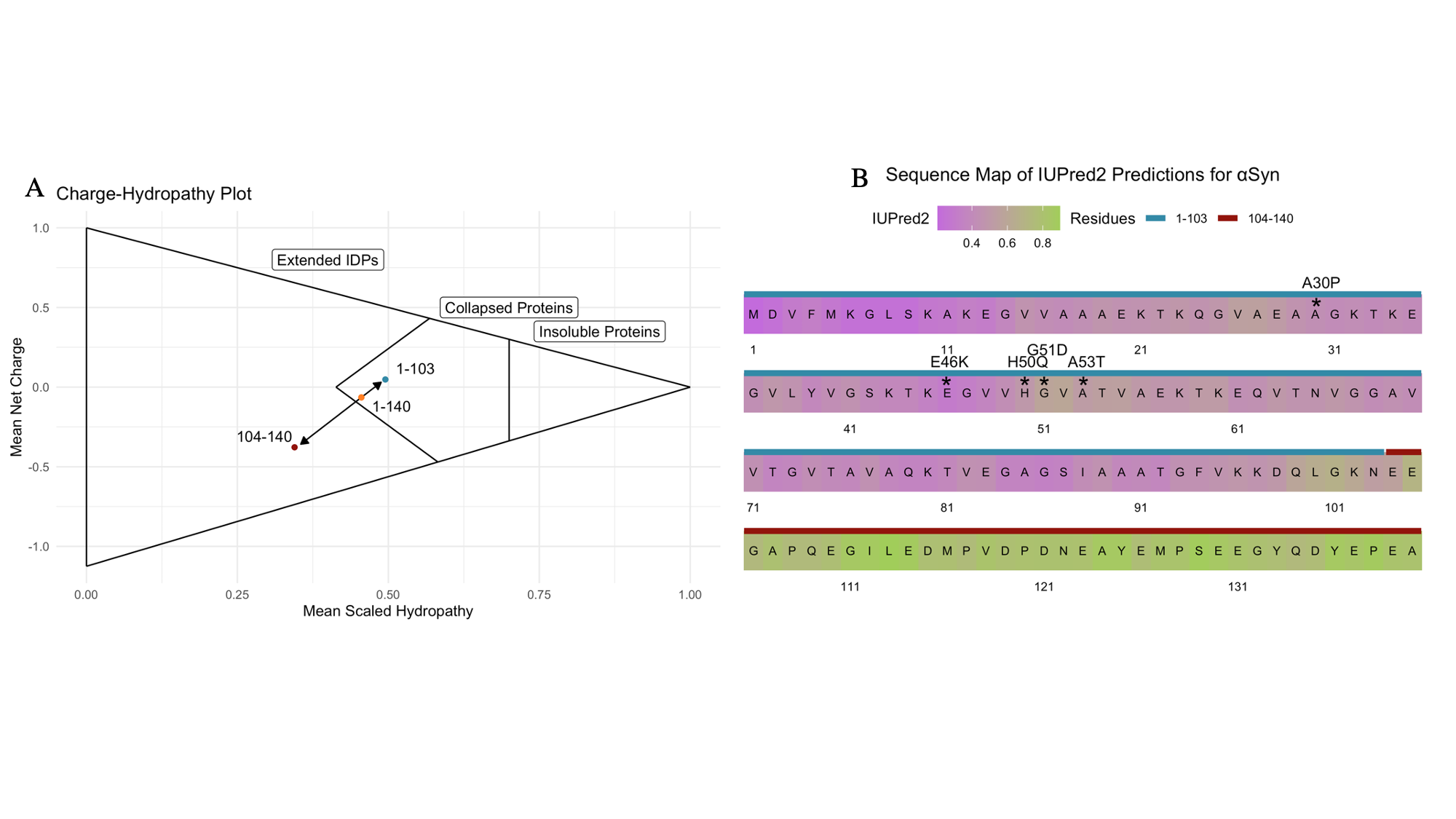
**

**Fig S1 – α-Synuclein C-terminal is predicted as unfolded.**

Plots generated using *idpr* and further customized with ggplot2 annotations [1].

(A) Charge-hydropathy plot of truncated α-Synuclein (αSyn; UniProt ID: P37840) sequences predicts a collapsed protein and an IDR in regions 104-140. Points colored by αSyn residues: 1-140 (orange), 1-103 (blue), 104-140 (red). Method described in [2]. Mean Scaled Hydropathy calculated with The Kyte and Doolittle measurement of hydropathy [3], scaled to Arg = 0 and Ile =1. Mean Net Charge calculated with IPC_protein pKa values [4]. Cutoff equation is <Charge>=±2.785<Hydropathy>±1.151 as described previously [5]. Proteins are considered insoluble when <Hydropathy> ≥ 0.7.

(B) Sequence Map of IUPred2 Prediction of Disorder for αSyn.

IUPred2 predicts a C-terminal IDR in αSyn [6, 7]. Residues with a score 0.0-0.5 are predicted to be ordered, regions 0.5-1.0 are predicted to be disordered. The bar above indicates analyzed αSyn domains: residues 1-103 (blue), residues 104-140 (red). Mutations associated with familial Parkinson’s Disease are indicated [8-10]. *idpr* is available at <https://www.bioconductor.org/packages/idpr>


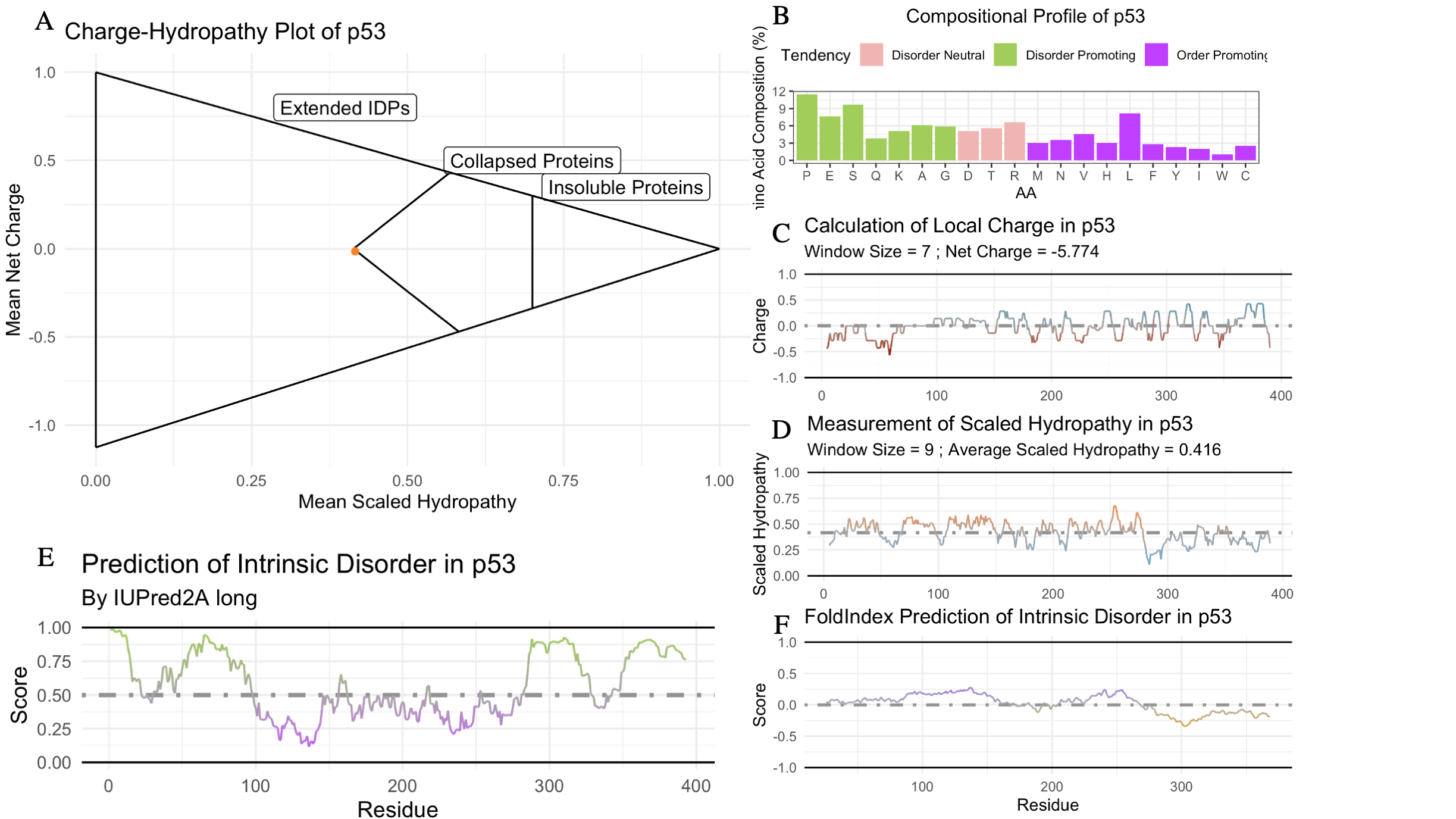


**Fig S2 – The idprofile of p53, generated by *idpr*, returns IDP characteristics.**

(A) Charge-hydropathy plot of p53 (UniProt ID: P04637) predicts the sequence is an extended IDP. Method described in [2]. Mean Scaled Hydropathy calculated with The Kyte and Doolittle measurement of hydropathy [3], scaled to Arg = 0 and Ile = 1. Mean Net Charge calculated with IPC_protein pKa values [4]. Cutoff equation is <Charge>=±2.785<Hydropathy>±1.151 as described previously [5]. Insoluble when <Hydropathy> ≥ 0.7.

(B) Structural tendency plot shows an p53 is enriched in disorder-promoting residues. Disorder-promoting residues in green; order-promoting residues in purple; disorder‐neutral residues in pink [11].

(C) Local Charge Plot shows an acidic N-terminus and basic C-terminus. The local charge is the average of a 7 amino acid wide sliding window, calculated with the IPC_protein pKa values [4].

(D) Local Hydropathy Plot shows an overall deficient in hydrophobic residues, especially at the C-terminus. The local hydropathy is the average of a 9 amino acid wide sliding window, calculated with the scaled Kyte and Doolittle measurement of hydropathy [3].

(E) IUPred2 predicts multiple IDRs [6, 7]. Residues with a score 0.0-0.5 are predicted to be ordered, regions 0.5-1.0 are predicted to be disordered.

(F) FoldIndex predictions disorder in p53 [12]. Residues with a score 0.0 – +1.0 are predicted to be ordered, regions -1.0 – 0.0 are predicted to be disordered.

**Supplementary Document References**

1. Wickham H. ggplot2: elegant graphics for data analysis. New York: Springer-Verlag; 2016.

2. Uversky VN, Gillespie JR, Fink AL. Why are “natively unfolded” proteins unstructured under physiologic conditions? Proteins: Structure, Function, and Bioinformatics. 2000;41(3):415-27.

3. Kyte J, Doolittle RF. A simple method for displaying the hydropathic character of a protein. Journal of molecular biology. 1982;157(1):105-32.

4. Kozlowski LP. IPC – Isoelectric Point Calculator. Biology Direct. 2016;11(1):55.

5. Uversky VN. Intrinsically Disordered Proteins and Their “Mysterious” (Meta)Physics. Frontiers in Physics. 2019;7(10).

6. Mészáros B, Erdős G, Dosztányi Z. IUPred2A: context-dependent prediction of protein disorder as a function of redox state and protein binding. Nucleic acids research. 2018;46(W1):W329-W37.

7. Erdős G, Dosztányi Z. Analyzing Protein Disorder with IUPred2A. Current Protocols in Bioinformatics. 2020;70(1):e99.

8. Kasten M, Klein C. The many faces of alpha‐synuclein mutations. Movement Disorders. 2013;28(6):697-701.

9. Flagmeier P, Meisl G, Vendruscolo M, Knowles TPJ, Dobson CM, Buell AK, et al. Mutations associated with familial Parkinson’s disease alter the initiation and amplification steps of α-synuclein aggregation. Proceedings of the National Academy of Sciences. 2016;113(37):10328.

10. Fujioka S, Ogaki K, Tacik PM, Uitti RJ, Ross OA, Wszolek ZK. Update on novel familial forms of Parkinson's disease and multiple system atrophy. Parkinsonism & Related Disorders. 2014;20:S29-S34.

11. Uversky VN. Unusual biophysics of intrinsically disordered proteins. Biochimica et Biophysica Acta (BBA) - Proteins and Proteomics. 2013;1834(5):932-51.

12. Prilusky J, Felder CE, Zeev-Ben-Mordehai T, Rydberg EH, Man O, Beckmann JS, et al. FoldIndex©: a simple tool to predict whether a given protein sequence is intrinsically unfolded. Bioinformatics. 2005;21(16):3435-8.
