# Supplementary material for: idpr: A package for profiling and analyzing Intrinsically Disordered Proteins in R
Source: PLoS One. 2022 Apr 18;17(4):e0266929. doi: 10.1371/journal.pone.0266929 (PMC9015136; doi:10.1371/journal.pone.0266929)
Supplement: S2 File — (PDF) [file pone.0266929.s002.pdf]

idpr Workflow

William McFadden & Judith Yanowitz

3/27/2022

Contents

1 Installing idpr **2**

1.1 Downloading the Current Release 2

1.2 Downloading the Development Version 2

1.3 Loading idpr 2

1.4 workflow-only dependency 3

2 alpha-Synuclein Figures **3**

2.1 Fetching the amino acid sequence 3

2.2 Generating the idprofile for alpha-Synuclein (Fig. 1) 3

2.3 Generating Supplemental Figures for alpha-Synuclein 9

3 p53 Figures **13**

3.1 Fetching the amino acid sequence 13

3.2 Fetching IUPred2A (Fig. 2A) 14

3.3 Fetching IUPred2 Redox (Fig. 2B) 14

3.4 Making the sequenceMap of p53's sequence structural tendency (Fig. 2C) 15

3.5 Generating the idprofile for p53 (Fig. S2) 17

4 GCNA Figures **23**

4.1 Fetching the amino acid sequence 23

4.2 Generating the idprofile for GCNA (Fig. 3) 23

5 Appendix **29**

5.1 References 29

5.2 Additional Information 32

The following code generates the figures within the manuscript entitled 'idpr: A Package for Profiling and Analyzing **I**ntrinsically **D**isordered **P**roteins in **R**.' by William McFadden and Judith Yanowitz.

1 Installing idpr

idpr is published in Bioconductor where the stable, released version of the package can be downloaded. The development version, which may be unstable, is available on GitHub.

Please note: To return all plots displayed in the aforementioned manuscript, you must run **idpr** v. 1.5.11 or later. **idpr** v. 1.6.0 will be released with the Bioconductor 3.15 release.

1.1 Downloading the Current Release

The package can be installed from Bioconductor with the following line of code. This requires the BiocManager package to be installed.

```
if(!'BiocManager' %in% installed.packages()) {
    install.packages("BiocManager")
}

if(!'idpr' %in% installed.packages()) {
    BiocManager::install("idpr")
}
```

1.2 Downloading the Development Version

The most recent version of the package can be installed with the following line of code. This requires the devtools package to be installed. Please use this method if **idpr** version is <1.5.11 .

```
if(!'devtools' %in% installed.packages()) {
    install.packages("devtools")
}

if(!'idpr' %in% installed.packages()) {
    devtools::install_github("wmm27/idpr")
}
```

1.3 Loading idpr

Once installed, **idpr** can be loaded with the 'library' function.

```
library(idpr)
```

To test the package is loaded, the **idpr** function 'netCharge' will be used to determine the charge of Glutamic Acid (E) at pH 8. Since pH » pKa, the charge of E should be near -1.

```
netCharge("E",
          pH = 8,
          includeTermini = FALSE)
```

```
## [1] -0.9997418
```

1.4 workflow-only dependency

The **UniprotR** package is used within this workflow to fetch the amino acid sequences for the proteins analyzed. **idpr** contains multiple ways to read in sequences, including from .fasta files *via* **Biostrings**. To avoid distributing additional files, we are utilizing **UniprotR** to fetch sequences from the UniProt database. To run this workflow please install **UniprotR**. **UniprotR** is not a dependency of **idpr**, though this workflow exemplifies how the packages can be used together.

```
if(!'UniprotR' %in% installed.packages()) {
    install.packages("UniprotR")
}
```

2 alpha-Synuclein Figures

2.1 Fetching the amino acid sequence

First, I will use the **UniprotR** package to get the alpha-synuclein amino acid sequence from the UniProtID. For alpha-Synuclein, the ID is P37840.

```
## Please wait we are processing your accessions ...
```

Retrieved Sequence:

```
print(a_syn_seq)
```

```
## [1] "MDVFMKGLSKAKEGVVAAAEKTKQGVAEAAGKTKEGVLYVGSKTKEGVVHGVATVAEKTKEQVTNVGGAVVTGVTAVAQKTVEGAGSIAAATGFV[illegible]
```

2.2 Generating the idprofile for alpha-Synuclein (Fig. 1)

To get the 'idprofile', a simple function is needed with the sequence and Uniprot ID specified. This generates all plots in figure 1.

```
idprofile(sequence = a_syn_seq,
          uniprotAccession = "P37840",
          proteinName = "alpha-Synuclein")
```

```
## [[1]]
```

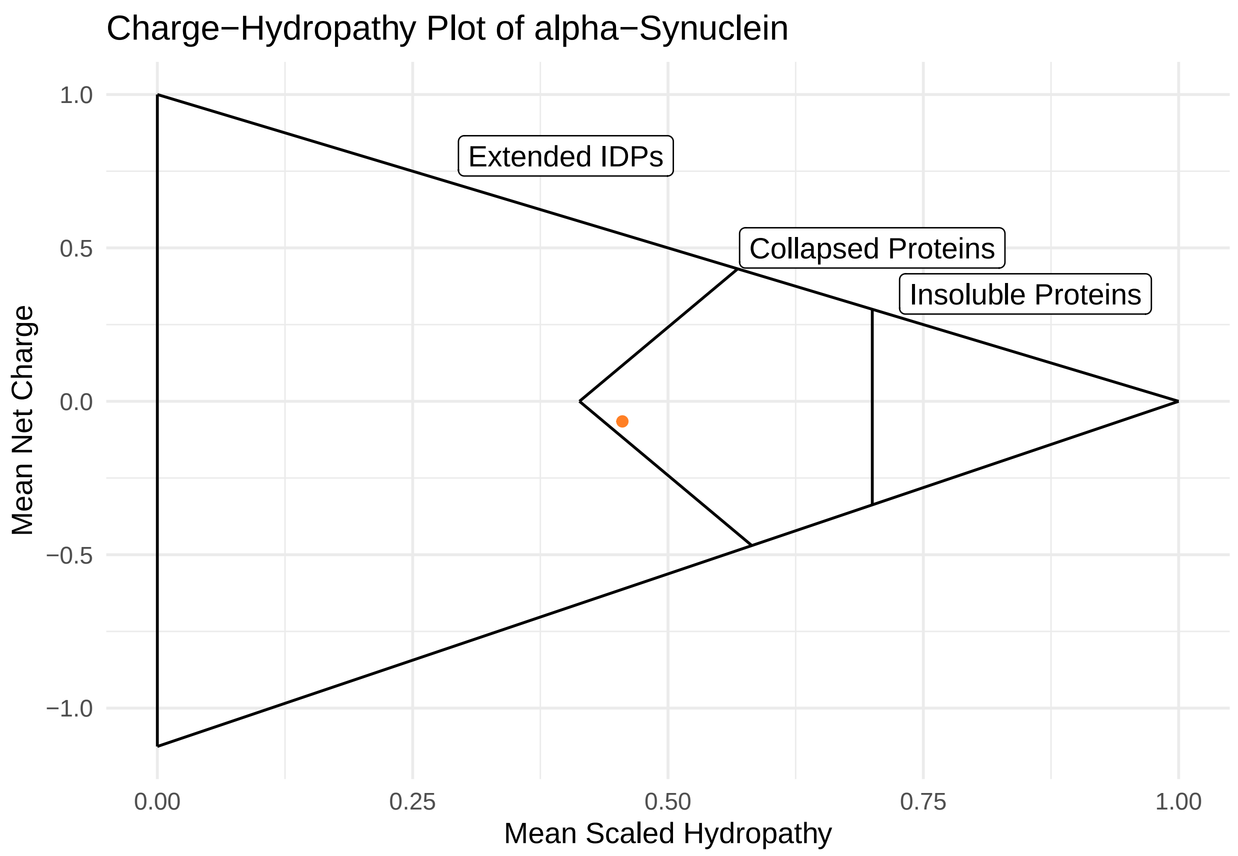
Charge−Hydropathy Plot of alpha−Synuclein
Extended IDPs
Collapsed Proteins
Insoluble Proteins
Mean Net Charge
1.0
0.5
0.0
−0.5
−1.0
0.00
0.25
0.50
0.75
1.00
Mean Scaled Hydropathy

```
##
## [[2]]
```

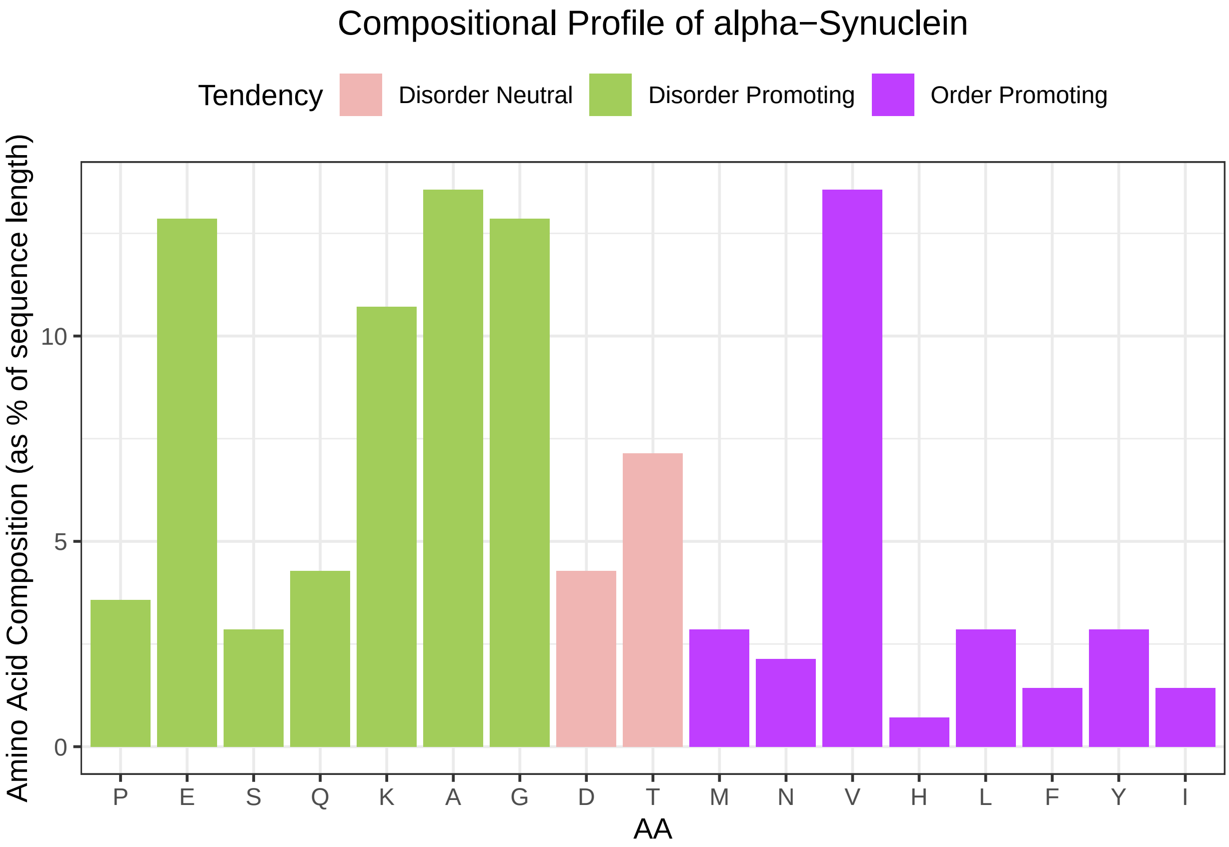
Compositional Profile of alpha−Synuclein
Tendency
Disorder Neutral
Disorder Promoting
Order Promoting
Amino Acid Composition (as % of sequence length)
0
5
10
P
E
S
Q
K
A
G
D
T
M
N
V
H
L
F
Y
I
AA

```
##
## [[3]]
```

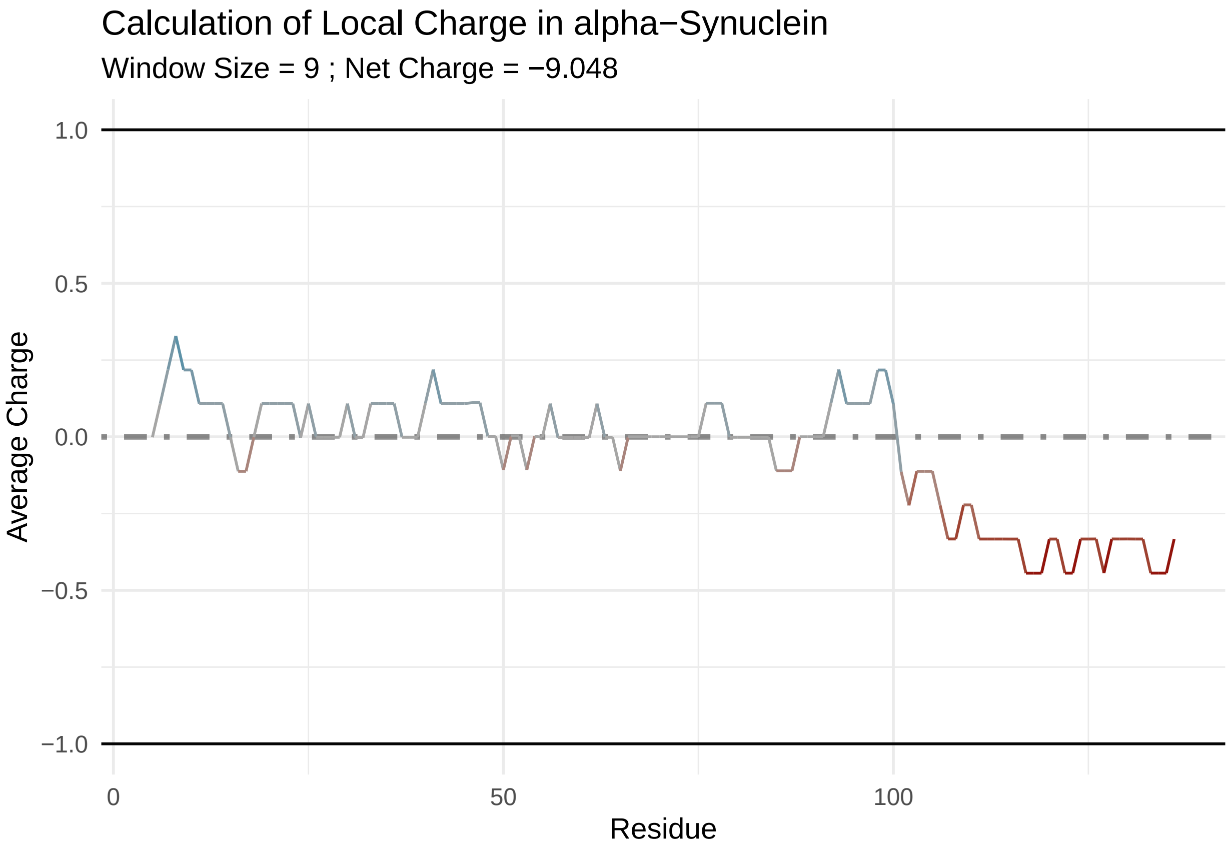
Calculation of Local Charge in alpha−Synuclein
Window Size = 9 ; Net Charge = −9.048
1.0
0.5
0.0
−0.5
−1.0
Average Charge
0
50
100
Residue

```
##
## [[4]]
```

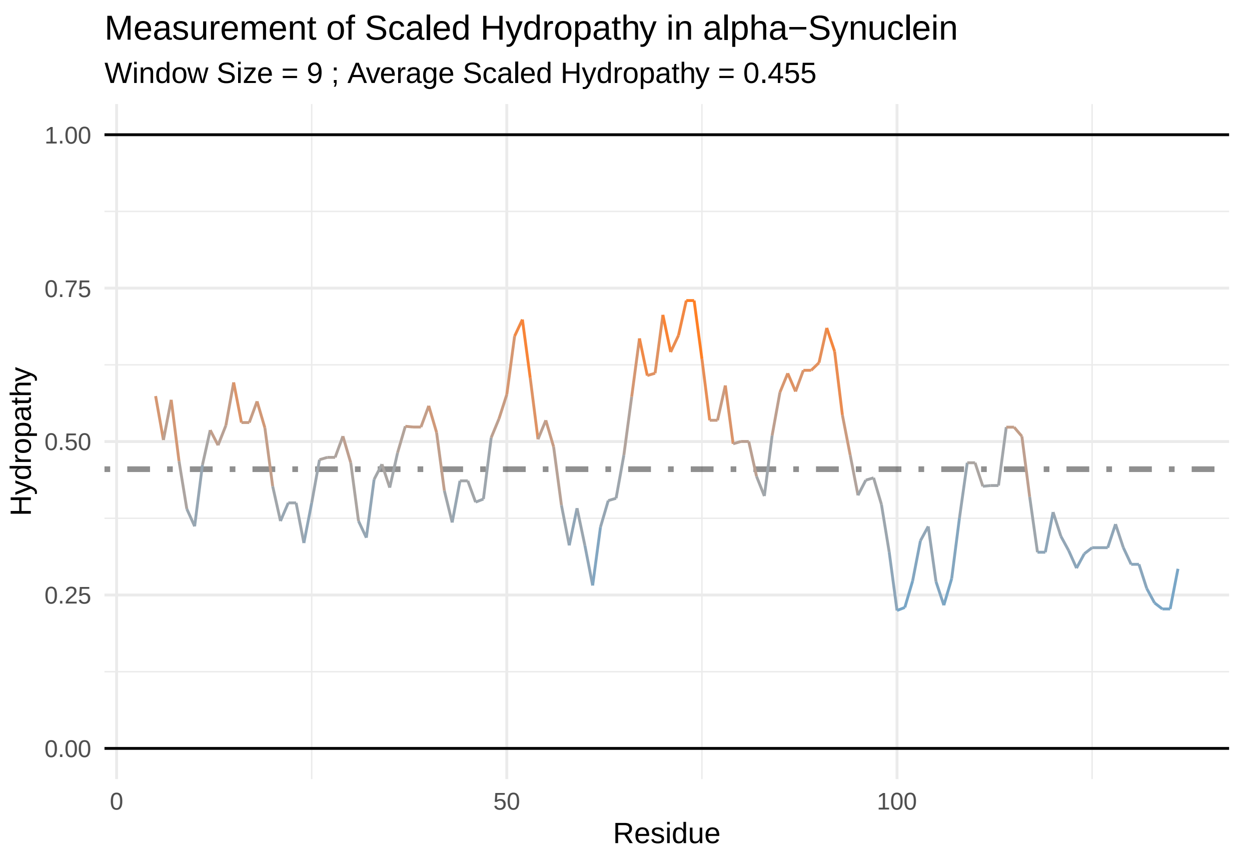
Measurement of Scaled Hydropathy in alpha−Synuclein
Window Size = 9 ; Average Scaled Hydropathy = 0.455
Hydropathy
1.00
0.75
0.50
0.25
0.00
0
50
100
Residue

```
##
## [[5]]
```

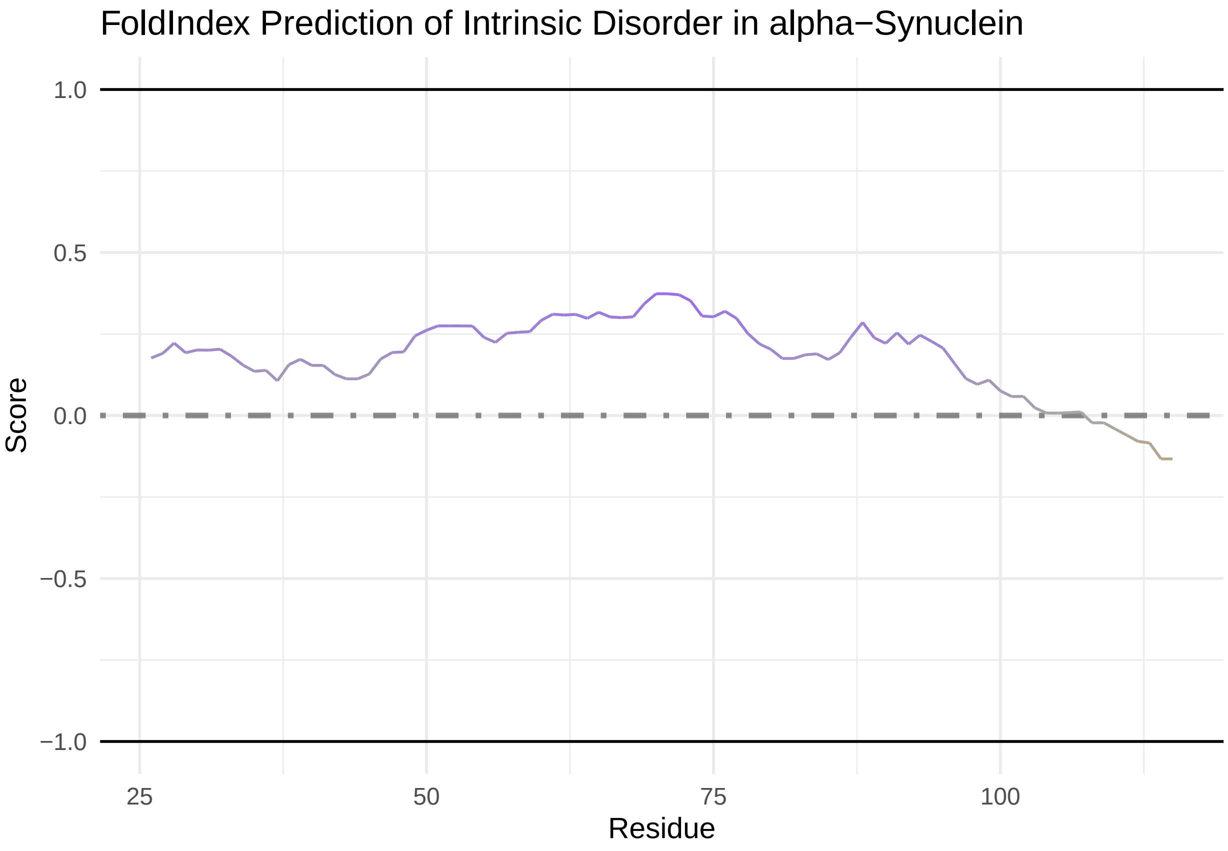

FoldIndex Prediction of Intrinsic Disorder in alpha−Synuclein
1.0
0.5
0.0
−0.5
−1.0
Score
25
50
75
100
Residue


```
##
## [[6]]
```

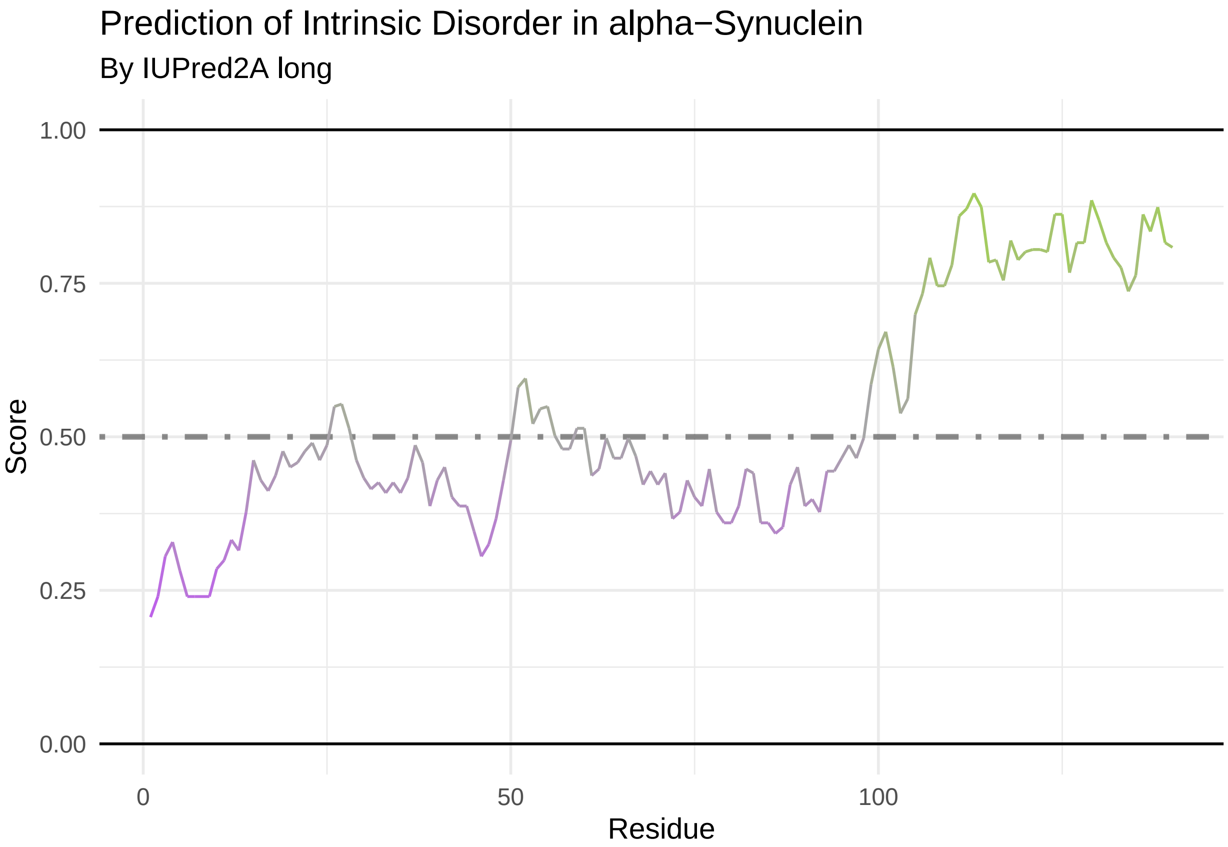

2.3 Generating Supplemental Figures for alpha-Synuclein

The following code generates plots for supplemental figure 1

2.3.1 Charge-Hydropathy plot of protein domains (Fig. S1A)

To add multiple points to the charge hydropathy plot, first the sequence will be split into the N-term (residues 1-103) and C-term (residues 104-140). To do this, I will use the 'AAString' function from **Biostrings**. For the split sequences and the full length sequence, the average net charge and the mean scaled hydropathy are calculated and put into a data frame. These coordinates will be used for adding **ggplot2** annotations. Since **idpr** depends on both of these packages, they should already be installed.

```
# --- Load packages
library(ggplot2)
library(Biostrings)
```

R	H	Name	Name_Expression
0.0478027	0.4950485	1-103	aSyn [1-103]
-0.3778272	0.3447297	104-140	aSyn [104-140]
-0.0646286	0.4553214	1-140	aSyn [1-140]

Then, the ggplot is made and annotations are added. See **ggplot2** for annotation options.

```r
# --- Make the base plot
a_syn_split_plot <- chargeHydropathyPlot(a_syn_seq_split)

# --- Add arrows to plot using ggplot2 geom_segment() function.
    # Arrows start at aSyn [1-140] and point to domains.
a_syn_split_plot <- a_syn_split_plot +
    geom_segment(aes(x = 0.4553214, y = -0.06462863, xend = 0.488, yend = 0.03),
                 arrow = arrow(length = unit(0.2, "cm"),
                               type = "closed"))+
    geom_segment(aes(x = 0.4553214, y = -0.06462863, xend = 0.355, yend = -0.358),
                 arrow = arrow(length = unit(0.2, "cm"),
                               type = "closed"))
# --- Add labels to points with ggplot2 functions
a_syn_split_plot <- a_syn_split_plot +
    geom_text(data = RH_DF,
              aes(x = H,
                  y = R,
                  label = Name),
               nudge_x = c(0.05, -0.05, 0.05),
               nudge_y = c(0.07, 0.070, -0.055)
    )
# --- Adds colored points to plot. Adds on top of geom_segment.
a_syn_split_plot <- a_syn_split_plot +
    geom_point(data = RH_DF,
              aes(x = H,
                  y = R),
              color = c("#348AA7", "#92140C", "chocolate1"))
```

```r
plot(a_syn_split_plot)
```

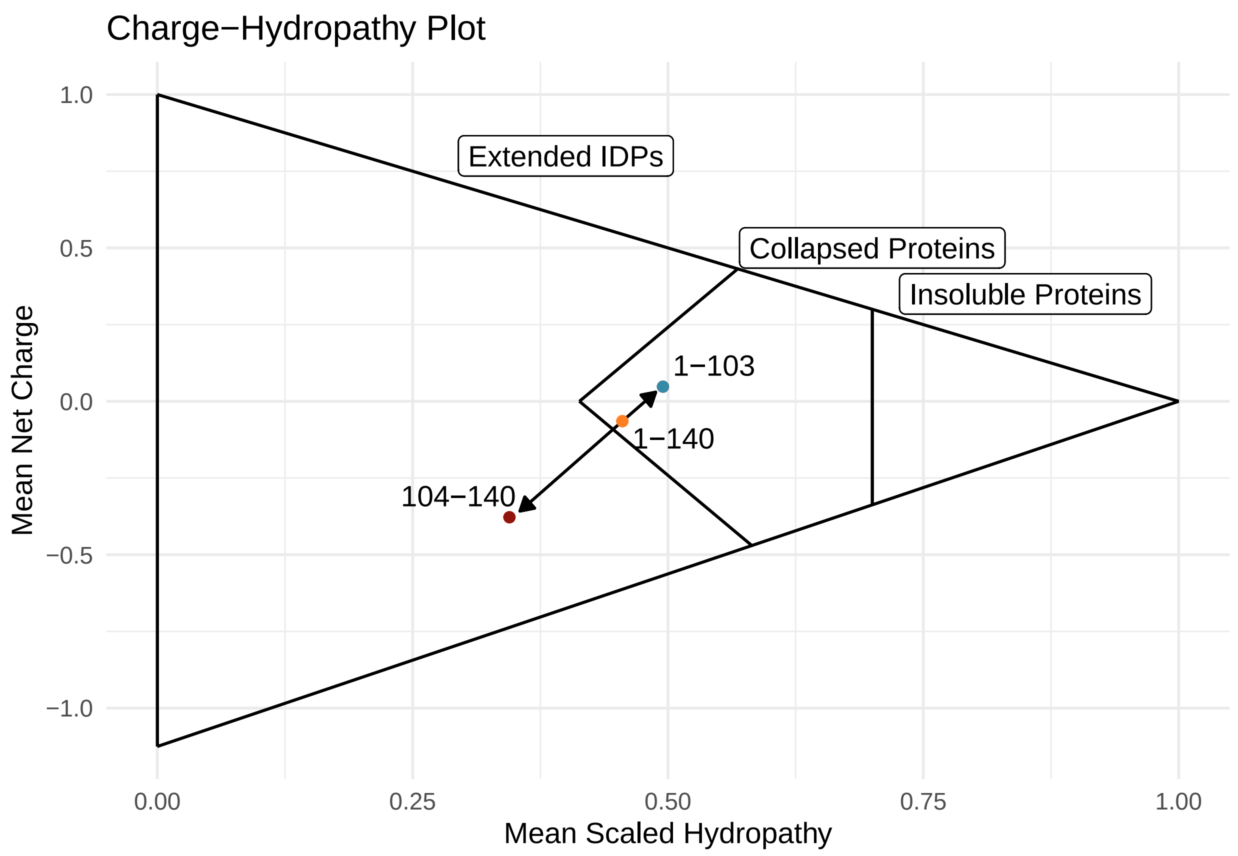

2.3.2 Sequence Map showing Familial Mutations (Fig. S1B)

Several point mutations in the alpha-Synuclein NTD have been identified that are linked to familial parkinsons disease. These are annotated here in the context of intrinsic disorder predictions from IUPred2. Functions from **ggplot2** are needed for annotations, and therefore this package must be attached if not already.

First, the data is retrieved from IUPred2. Setting plotResults = FALSE returns a data frame for custom plotting.

Position	AA	IUPred2
1	M	0.206376
2	D	0.239899
3	V	0.305330
4	F	0.328603
5	M	0.281712
6	K	0.239899

Then, a sequence map is created with the IUPred results. Column 2 (a_syn_iupred_df$AA) is a character vector of individual, single-letter amino acids. Column 3 (a_syn_iupred_df$IUPred2) is a numeric vector of IUPred2 scores.

```
iupred_map <-
    sequenceMap(sequence = a_syn_iupred_df$AA,
            property = a_syn_iupred_df$IUPred2,
            nbResidues = 35,
```

```r
            customColors = c("darkolivegreen3", "grey65", "darkorchid1"))
# --- Plot the unedited, unannotated sequenceMap
plot(iupred_map)
```

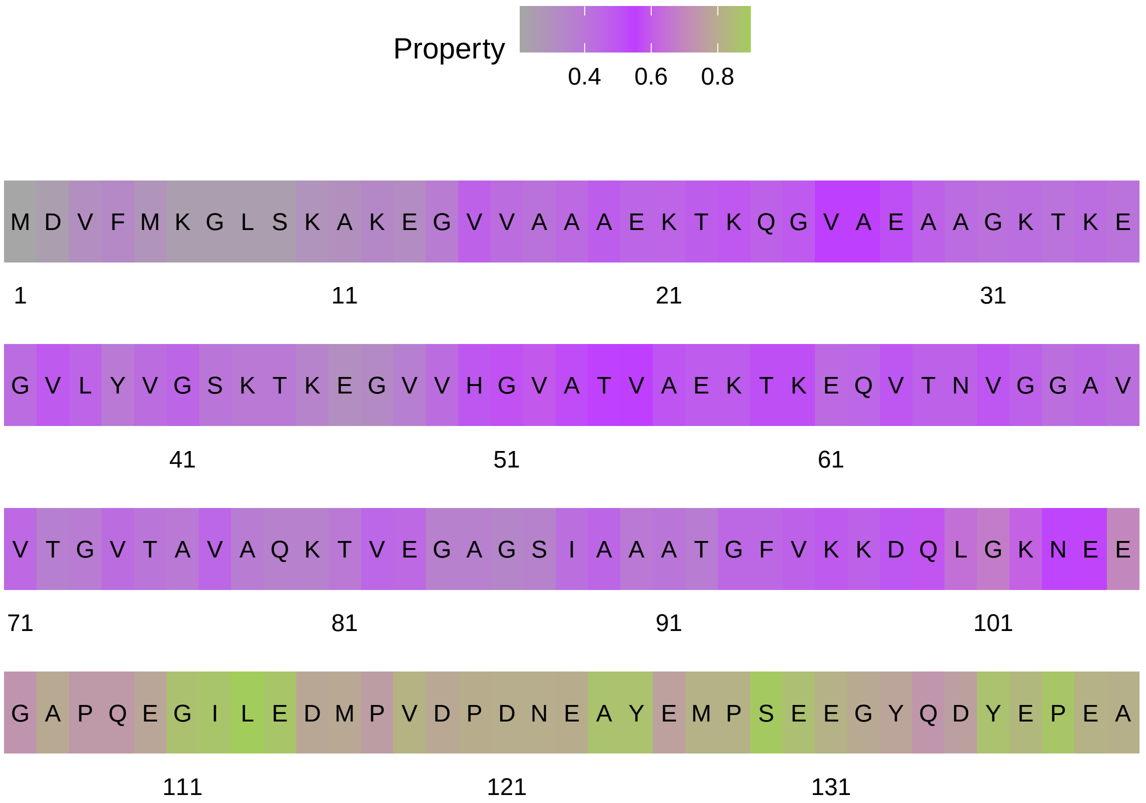

For adding annotations to a sequence map, you can get the position within the plot using the **idpr** function 'sequenceMapCoordinates'. This helps guide or identify the coordinates for **ggplot2** annotations.

Position	AA	row	col
1	M	4	1
2	D	4	2
3	V	4	3
4	F	4	4
5	M	4	5
6	K	4	6

Additionally, several annotations are added and the plot themes are edited. See code for all annotations.

Adding the labels for Familial Mutations and '*' to add above the mutated residues. These positions are determined by 'sequenceMapCoordinates' and values are added to row (y) value to move annotations above letters. To center residues, the column (x) values were adjusted by 0.5 or 0.35.

```r
iupred_map <- iupred_map +
    annotate("text",
             x = c(15.5, 18.5, 30.5, 11.5, 16.5),
             y = c(3.15, 3.15, 4.15, 3.15, 3.3),
             label = c("H50Q", "A53T", "A30P", "E46K", "G51D")) +
    annotate("text",
```

```
              x = c(15.35, 18.35, 30.25, 11.35, 16.35),
              y = c(2.825, 2.825, 3.825, 2.825, 2.825),
              label = rep("*", 5),
              size = 7)
```

Finally, the annotated sequence map is plotted.

```
plot(iupred_map)
```

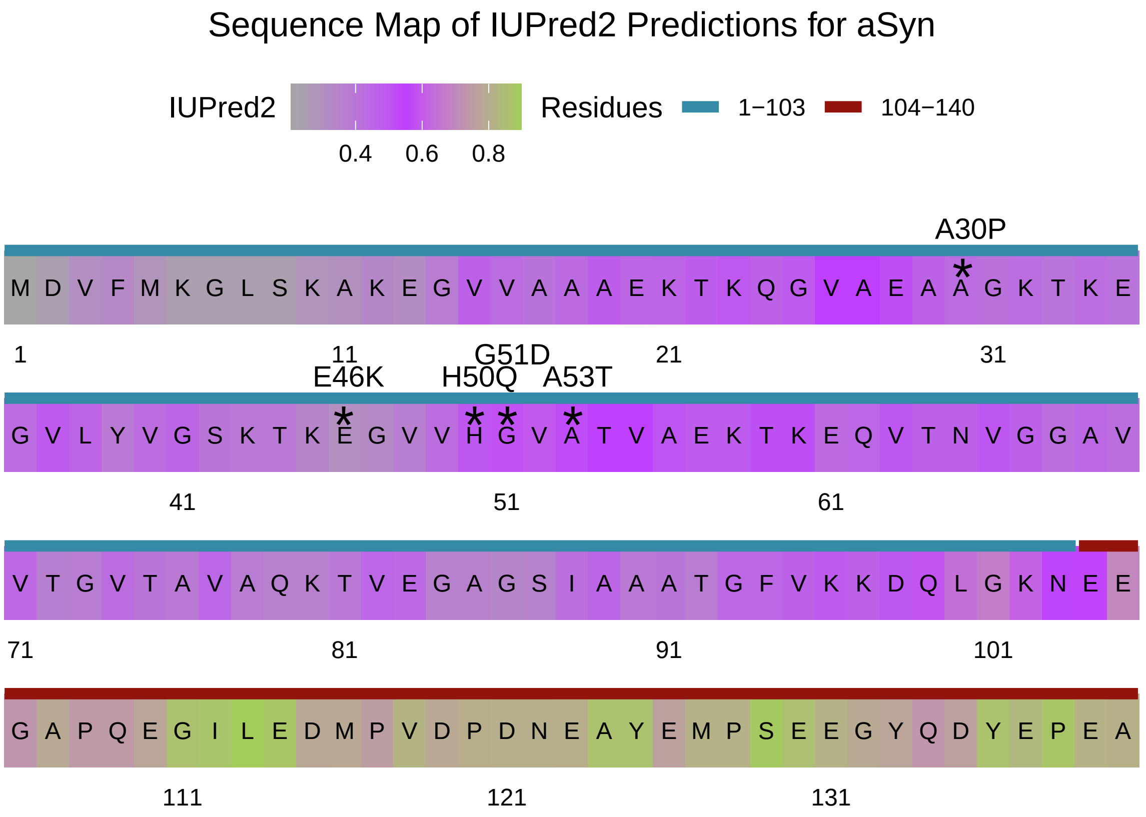

3 p53 Figures

3.1 Fetching the amino acid sequence

First, I will use the **UniprotR** package to get the p53 amino acid sequence from the UniProtID. For p53, the ID is P04637.

```
## Please wait we are processing your accessions ...
```

Retrieved Sequence:

```
print(p53_seq)
```

```
## [1] "MEEPQSDPSVEPPLSQETFSDLWKLLPENNVLSPLPSQAMDDLMLSPDDIEQWFTEDPGPDEAPRMPEAAPPVAPAPAAPTPAAPAPAPSWPLSS
```

3.2 Fetching IUPred2A (Fig. 2A)

To retrieve the IUPred2 long and ANCHOR2 scores, the p53 uniprot is used.

```
iupredAnchor(uniprotAccession = "P04637",
             proteinName = "p53")
```

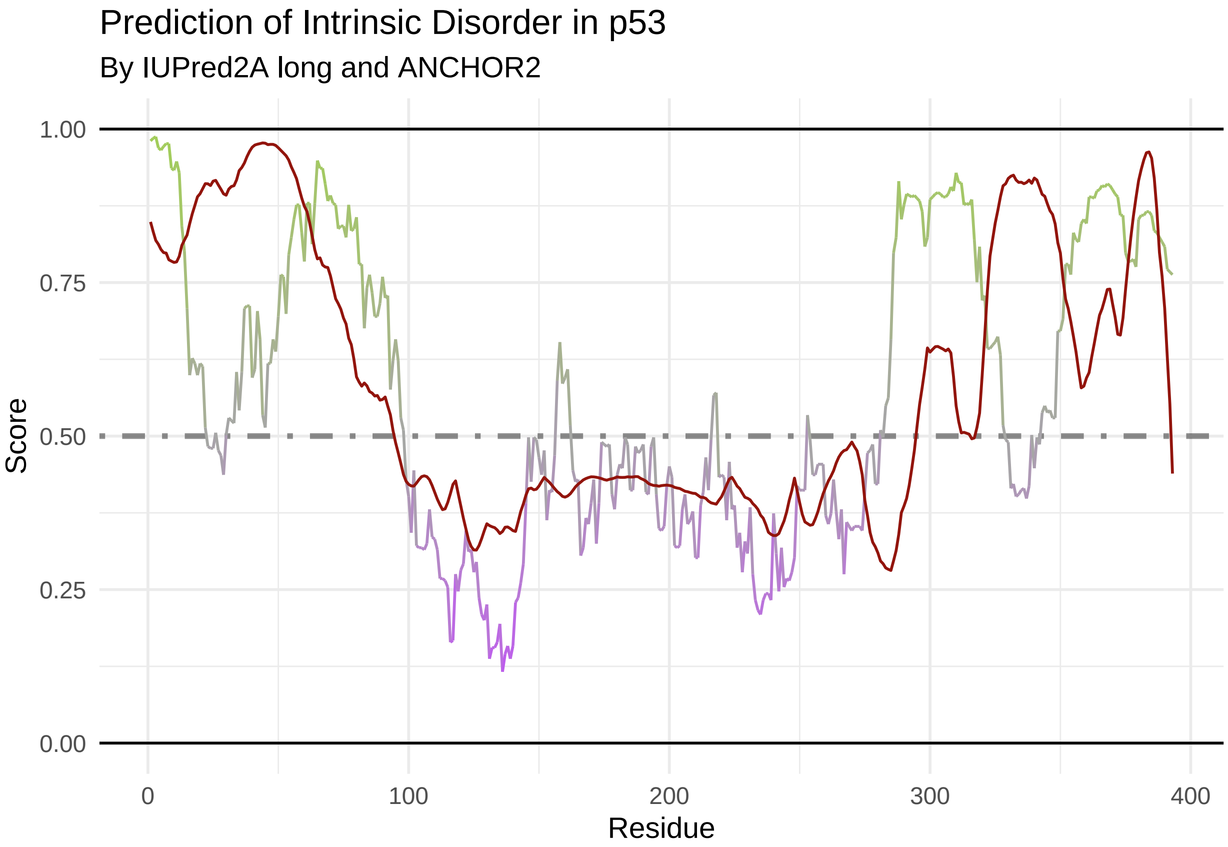

3.3 Fetching IUPred2 Redox (Fig. 2B)

To retrieve the IUPred2 with redox predictions, the p53 uniprot is used.

```
iupredRedox(uniprotAccession = "P04637",
            proteinName = "p53")
```

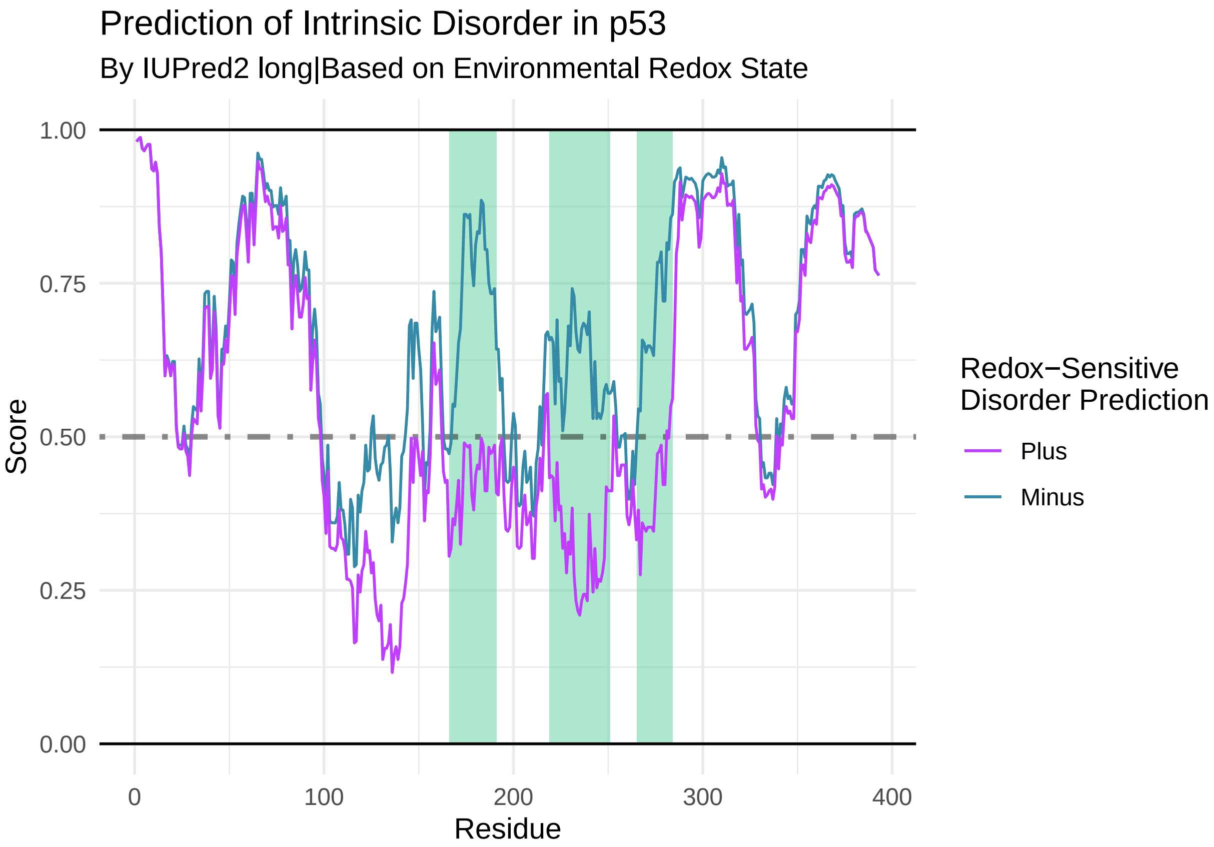

3.4 Making the sequenceMap of p53's sequence structural tendency (Fig. 2C)

First, the characteristic to map in the plot must be calculated. Here, the tendency for each residue to favor ordered/disordered structures is determined

```
tendency_DF <- structuralTendency(p53_seq)
knitr::kable(head(tendency_DF))
```

Position	AA	Tendency
1	M	Order Promoting
2	E	Disorder Promoting
3	E	Disorder Promoting
4	P	Disorder Promoting
5	Q	Disorder Promoting
6	S	Disorder Promoting

Then, the sequenceMap is made. Since p53 is a long sequence, the nbResidues are increased in the sequenceMap for easier viewing.

```
tendency_map <-
    sequenceMap(sequence = tendency_DF$AA,
            property = tendency_DF$Tendency,
            nbResidues = 79,
            customColors = c("#F0B5B3", "darkolivegreen3", "darkorchid1"))
```

```
        )
plot(tendency_map) #Return the unedited map
```

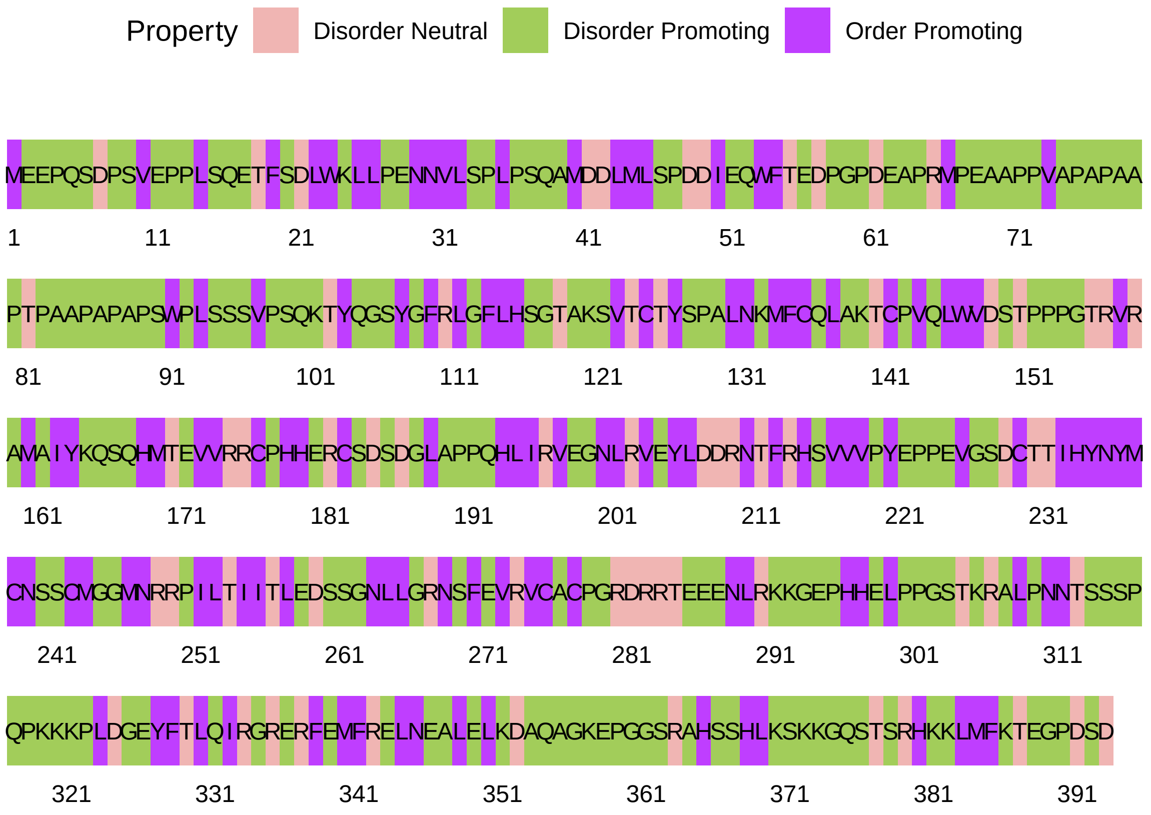

To get the coordinates for ggplot annotations, the 'sequenceMapCoordinates' function can assist. Since the default has been changed for nbResidues, from 30 to 79, this must change in the coordinates function to properly calculate the position of each residue within the sequenceMap.

```
p53_coords <- sequenceMapCoordinates(p53_seq,
                                      nbResidues = 79)
knitr::kable(head(p53_coords))  #Top of results to show example
```

Position	AA	row	col
1	M	5	1
2	E	5	2
3	E	5	3
4	P	5	4
5	Q	5	5
6	S	5	6

Additional annotations are made, see the code and the example from Fig. S1B on working with sequenceMap and annotations

After annotations and titles have been added, the plot can be generated

```
plot(tendency_map)
```

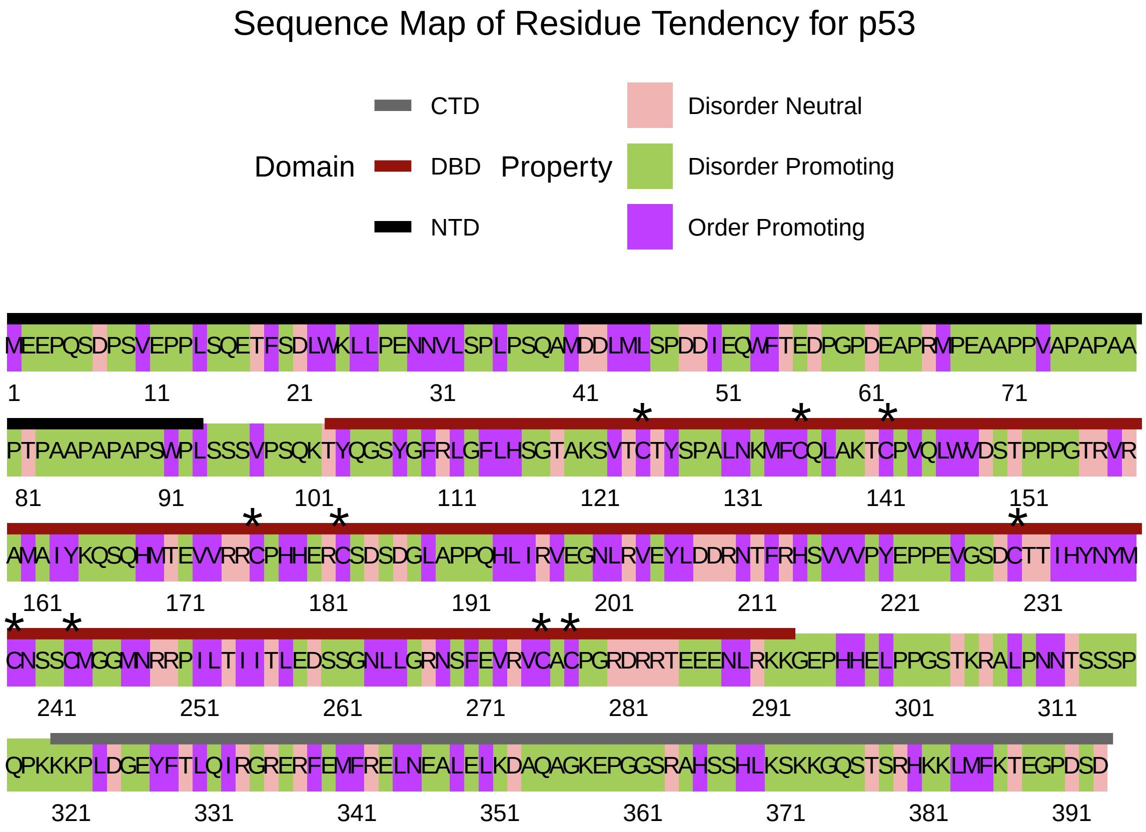

3.5 Generating the idprofile for p53 (Fig. S2)

To get the 'idprofile', a simple function is needed with the sequence and Uniprot ID specified. This generates all plots in supplementary figure 2.

```
idprofile(sequence = p53_seq,
          uniprotAccession = "P04637",
          proteinName = "p53") #Specifying proteinName automatically names plots
```

```
## [[1]]
```

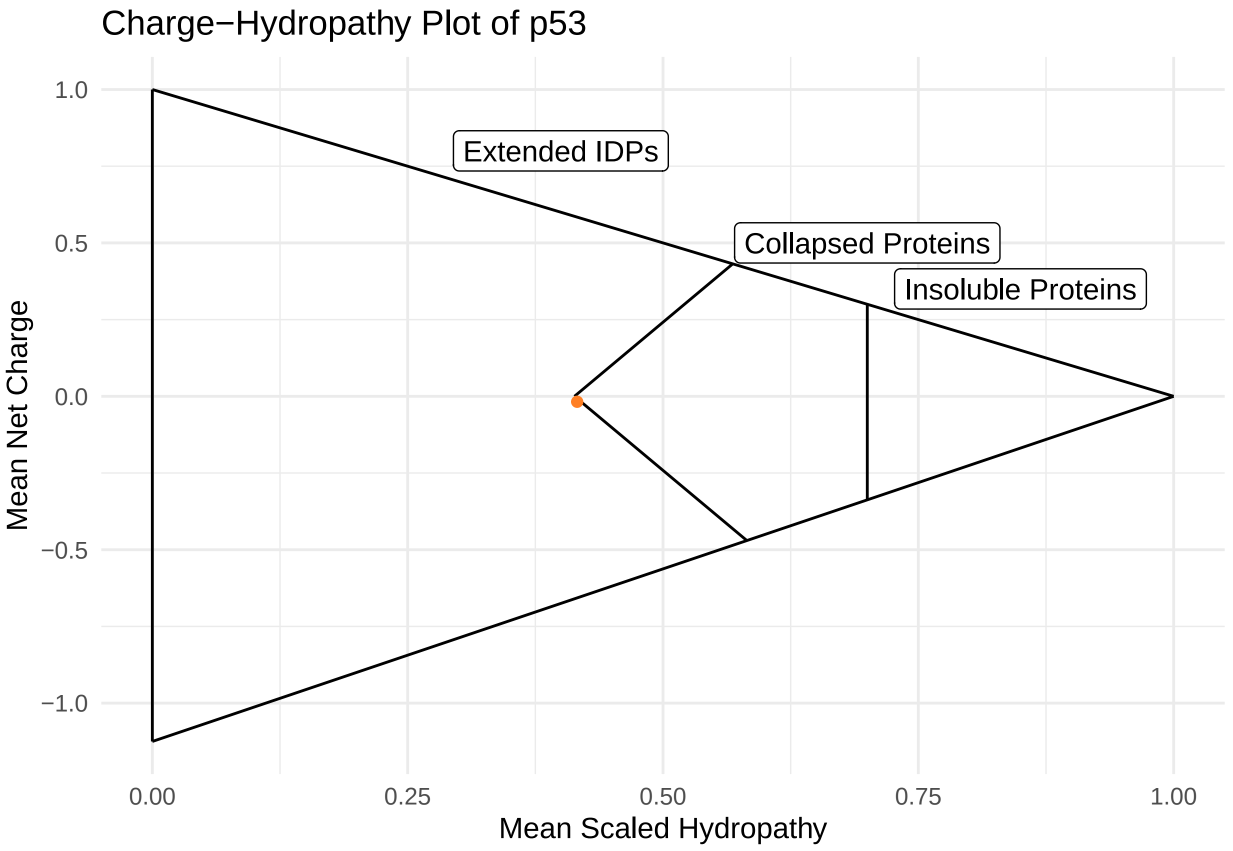
Charge−Hydropathy Plot of p53
Extended IDPs
Collapsed Proteins
Insoluble Proteins
Mean Net Charge
1.0
0.5
0.0
−0.5
−1.0
0.00
0.25
0.50
0.75
1.00
Mean Scaled Hydropathy

```
##
## [[2]]
```

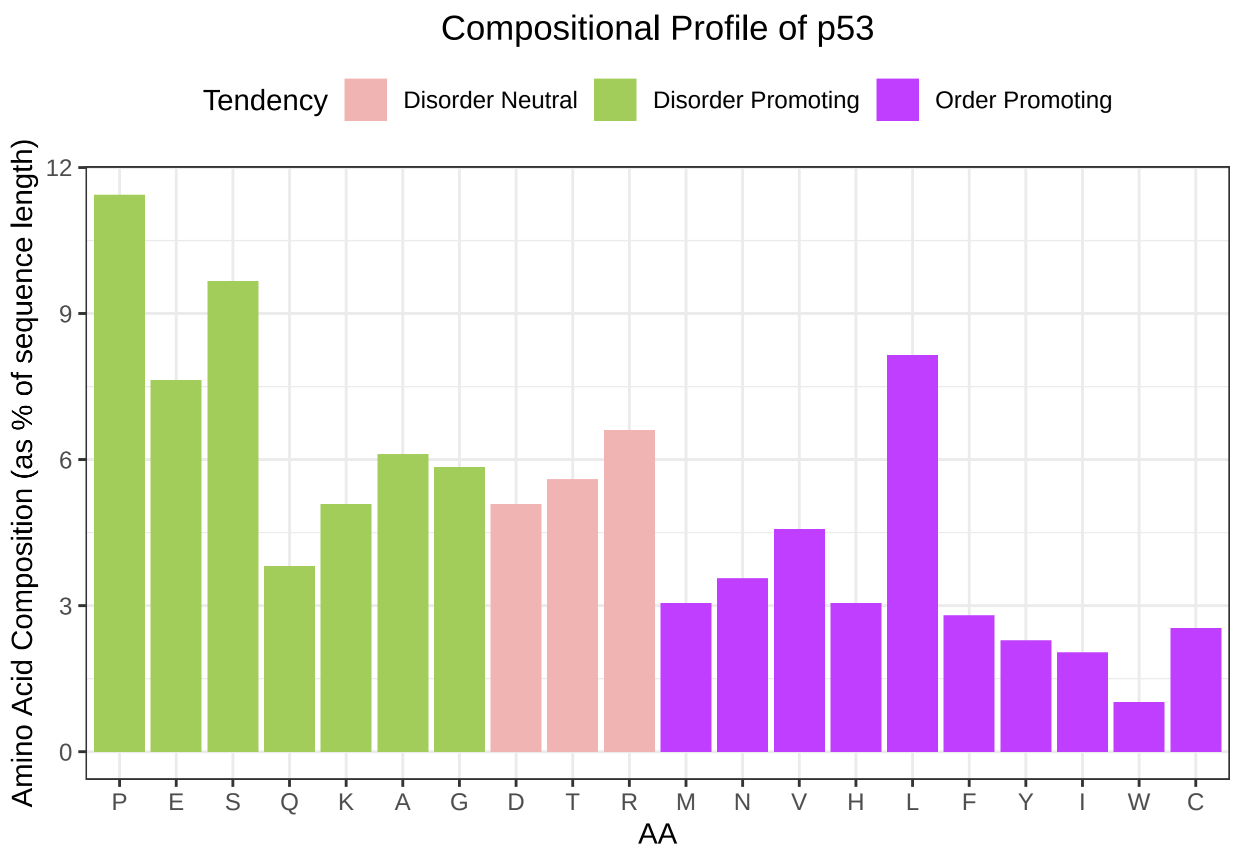
Compositional Profile of p53
Tendency
Disorder Neutral
Disorder Promoting
Order Promoting
Amino Acid Composition (as % of sequence length)
12
9
6
3
0
P E S Q K A G D T R M N V H L F Y I W C
AA

```
##
## [[3]]
```

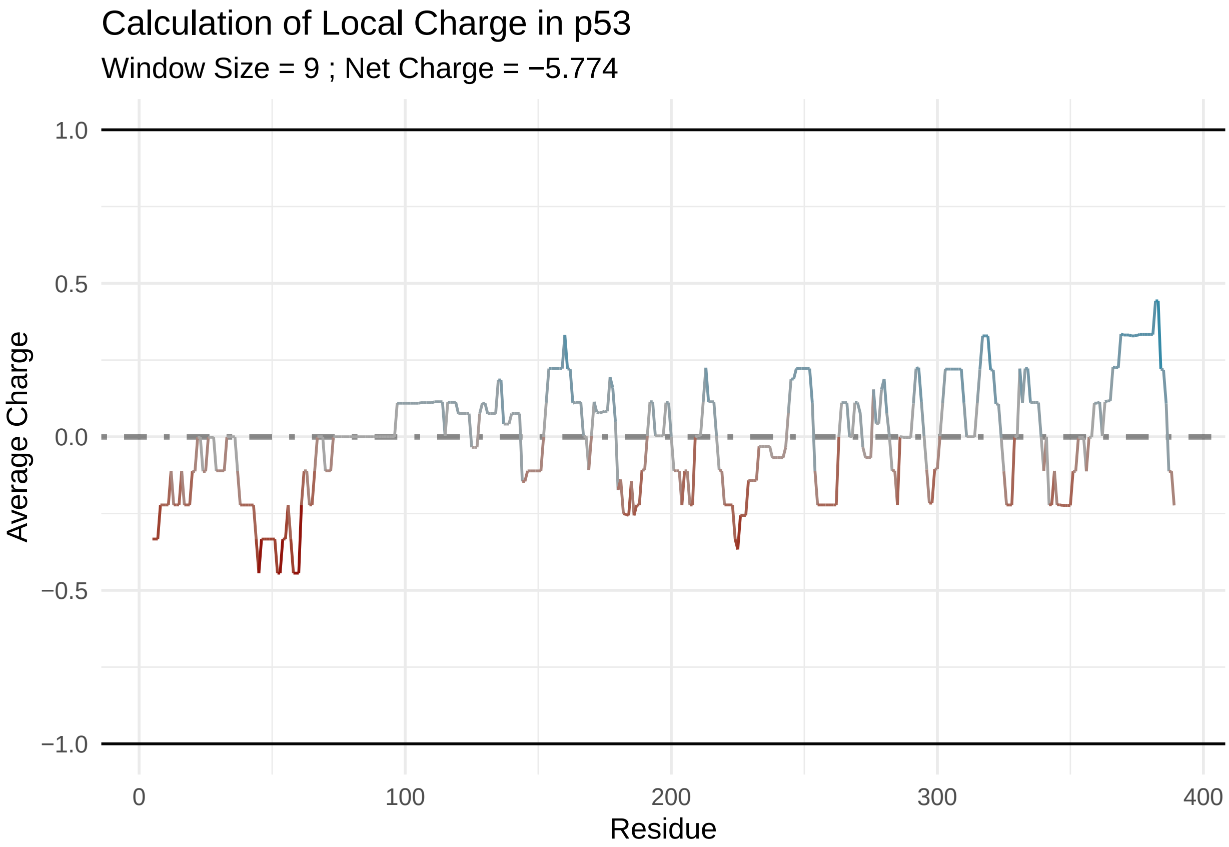
Calculation of Local Charge in p53
Window Size = 9 ; Net Charge = −5.774
Average Charge
1.0
0.5
0.0
−0.5
−1.0
0
100
200
300
400
Residue

```
##
## [[4]]
```

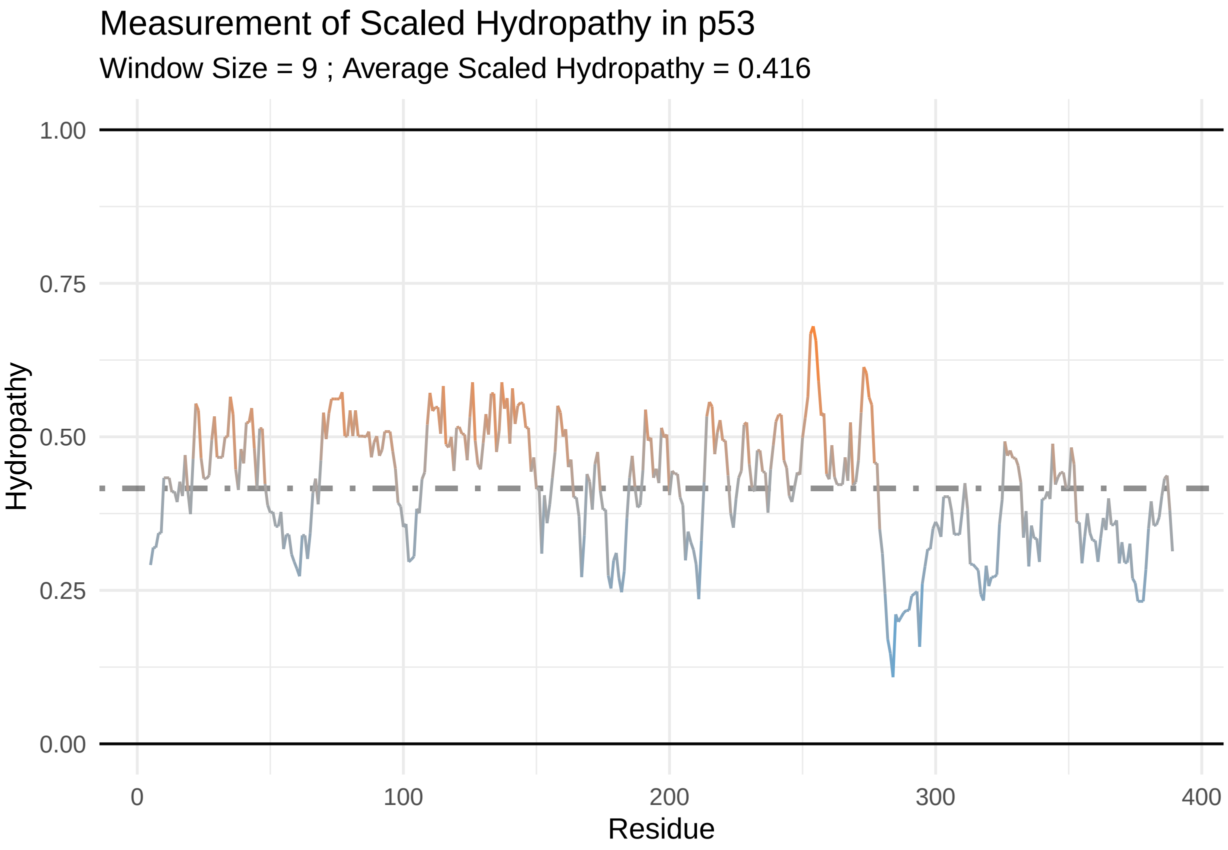
Measurement of Scaled Hydropathy in p53
Window Size = 9 ; Average Scaled Hydropathy = 0.416
1.00
0.75
0.50
0.25
0.00
Hydropathy
0
100
200
300
400
Residue

```
##
## [[5]]
```

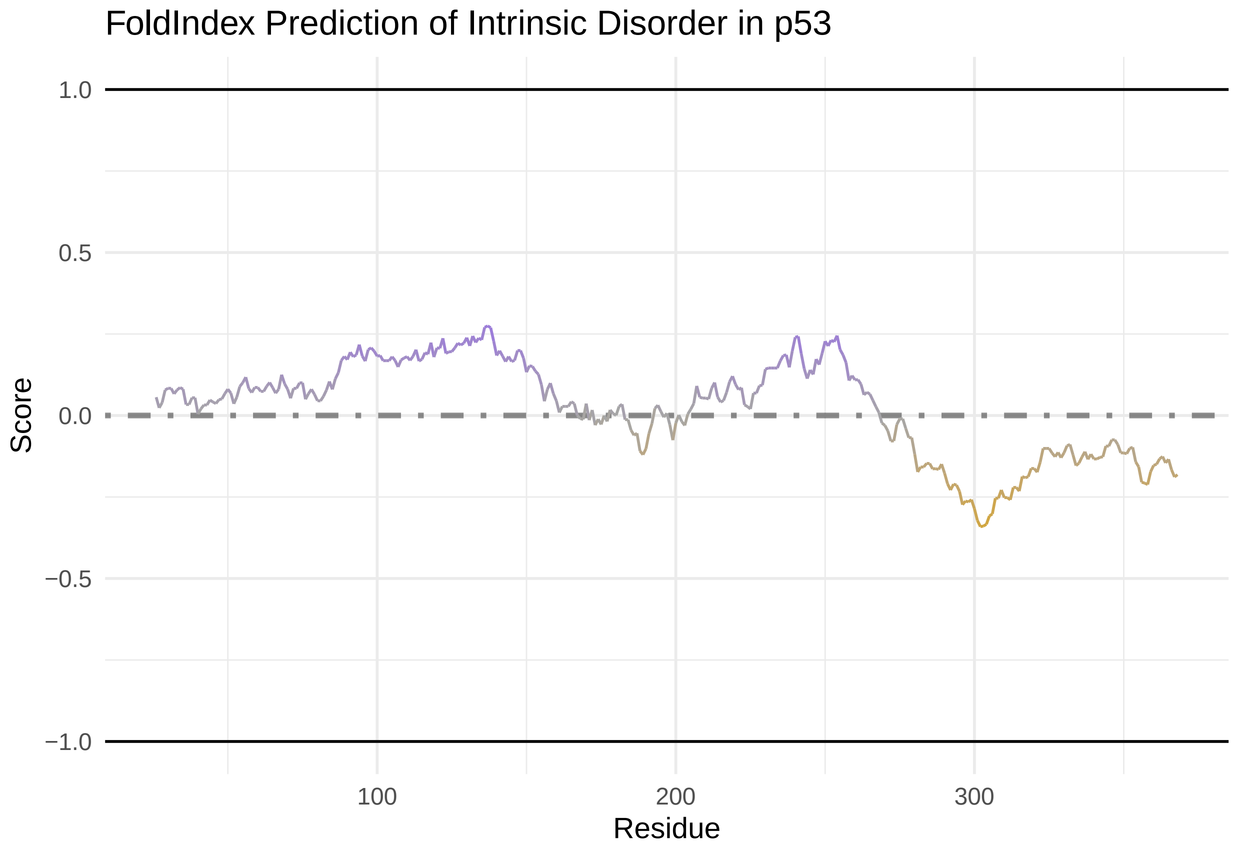
FoldIndex Prediction of Intrinsic Disorder in p53
Score
1.0
0.5
0.0
−0.5
−1.0
100
200
300
Residue

```
##
## [[6]]
```

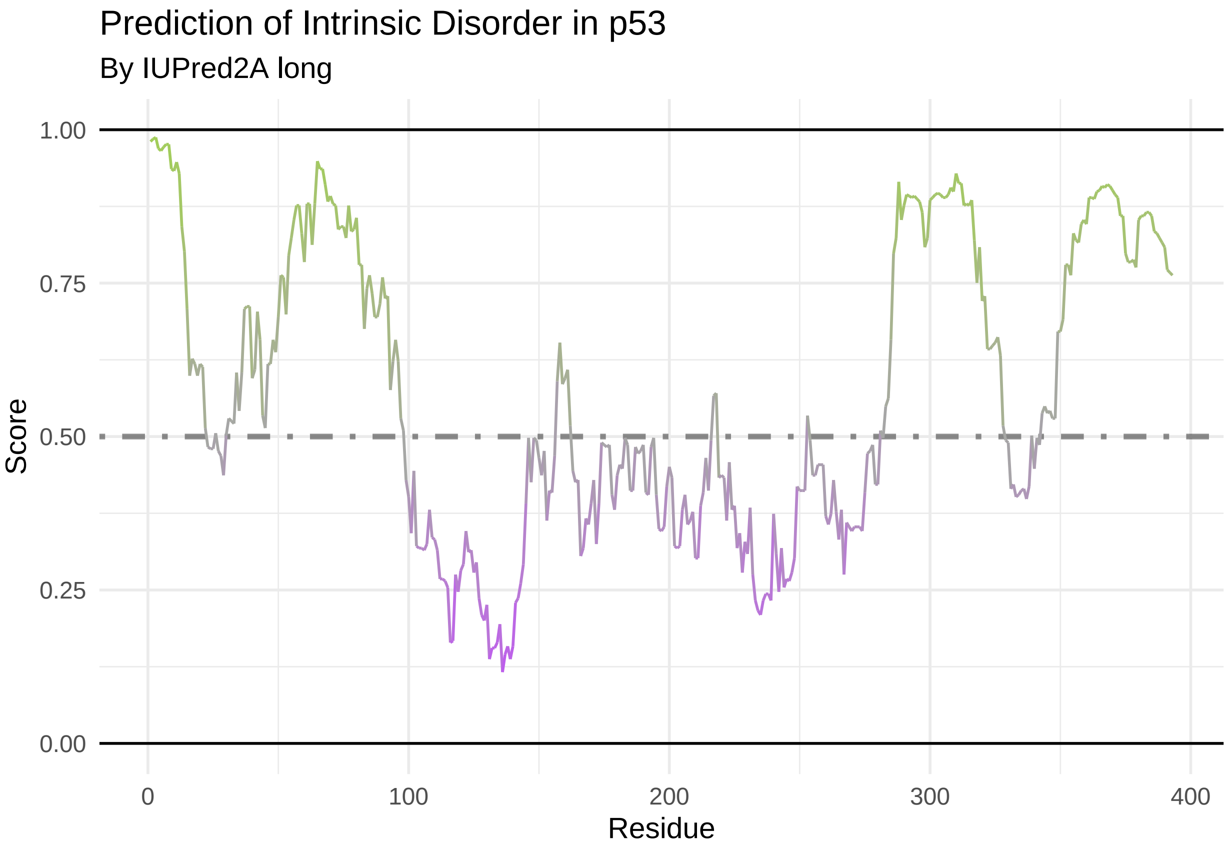

4 GCNA Figures

4.1 Fetching the amino acid sequence

First, I will use the **UniprotR** package to get the mouse (*M. musculus*) amino acid sequence from the UniProtID. For GCNA, the ID is A0A1D9BZF0.

```
## Please wait we are processing your accessions ...
```

Retrieved Sequence:

```
print(GCNA_seq)
```

```
## [1] "MDSGSSSSSSSSGSSSGSCSTSGSGSTSGSSTTSSSSSSSSSSSSSSSSSKEYMPELPKQRKASCVVIDSESDSDNTSDEKNTTVCEISSGDET
```

4.2 Generating the idprofile for GCNA (Fig. 3)

To get the ‘idprofile’, a simple function is needed with the sequence and Uniprot ID specified. This generates all plots in supplementary figure 3.

```
idprofile(sequence = GCNA_seq,
          uniprotAccession = GCNA_uniprot,
          proteinName = "Mouse GCNA")
```

```
## [[1]]
```

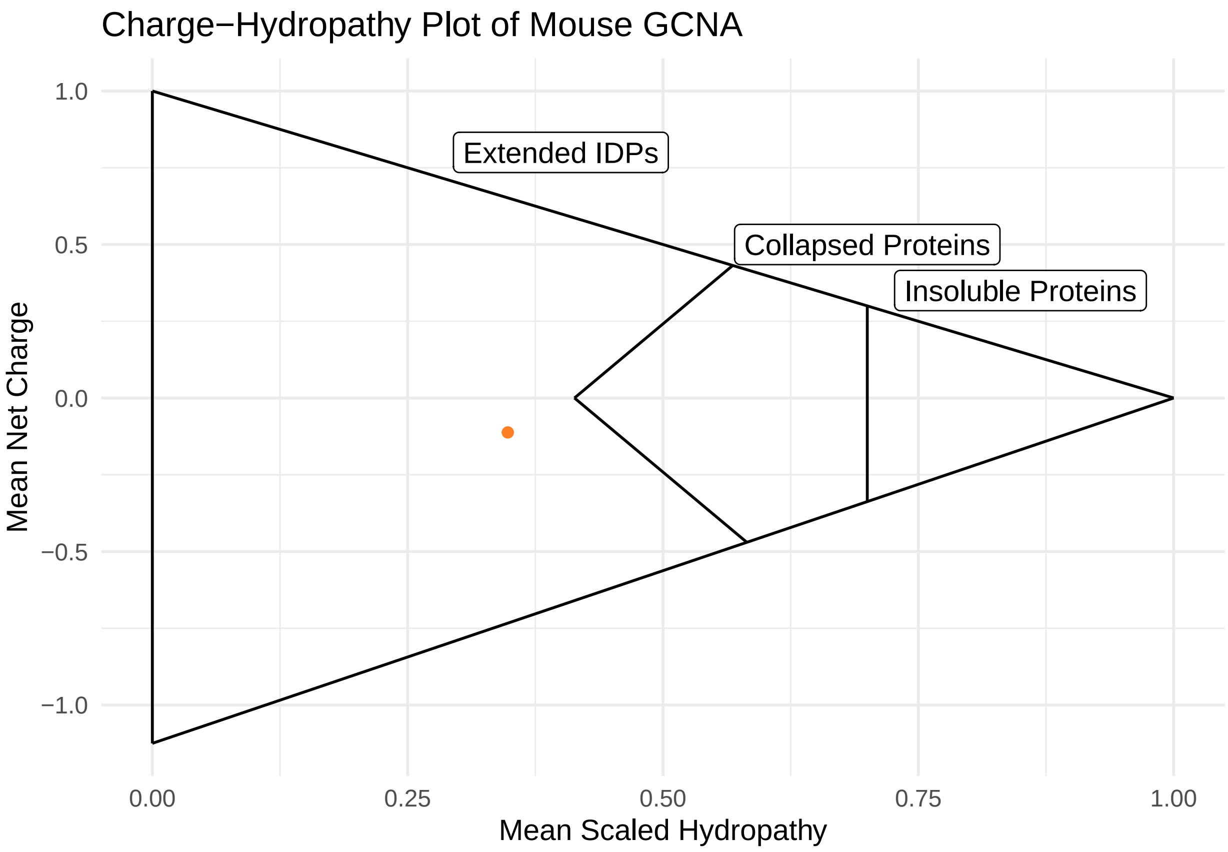

Charge−Hydropathy Plot of Mouse GCNA
Extended IDPs
Collapsed Proteins
Insoluble Proteins
Mean Net Charge
1.0
0.5
0.0
−0.5
−1.0
0.00
0.25
0.50
0.75
1.00
Mean Scaled Hydropathy


```
##
## [[2]]
```

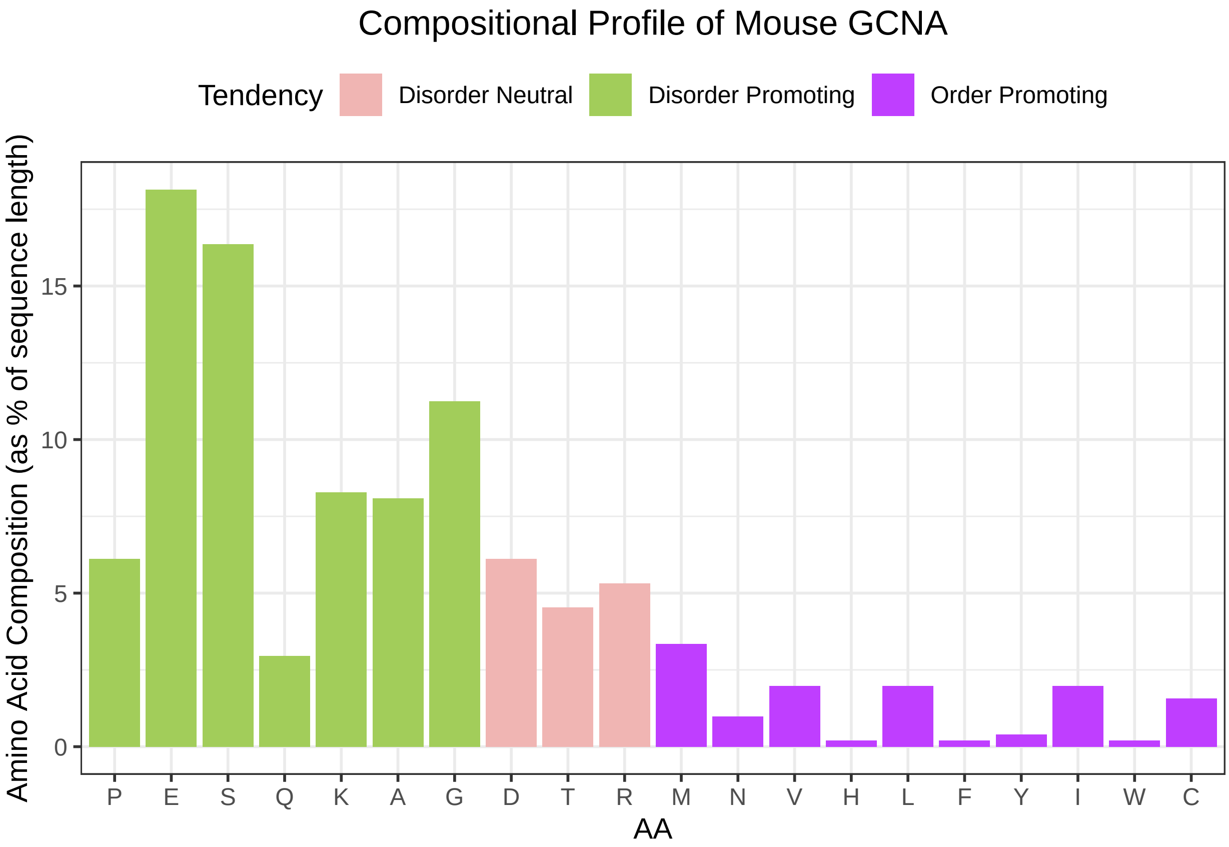
Compositional Profile of Mouse GCNA
Tendency
Disorder Neutral
Disorder Promoting
Order Promoting
Amino Acid Composition (as % of sequence length)
0
5
10
15
P E S Q K A G D T R M N V H L F Y I W C
AA

```
##
## [[3]]
```

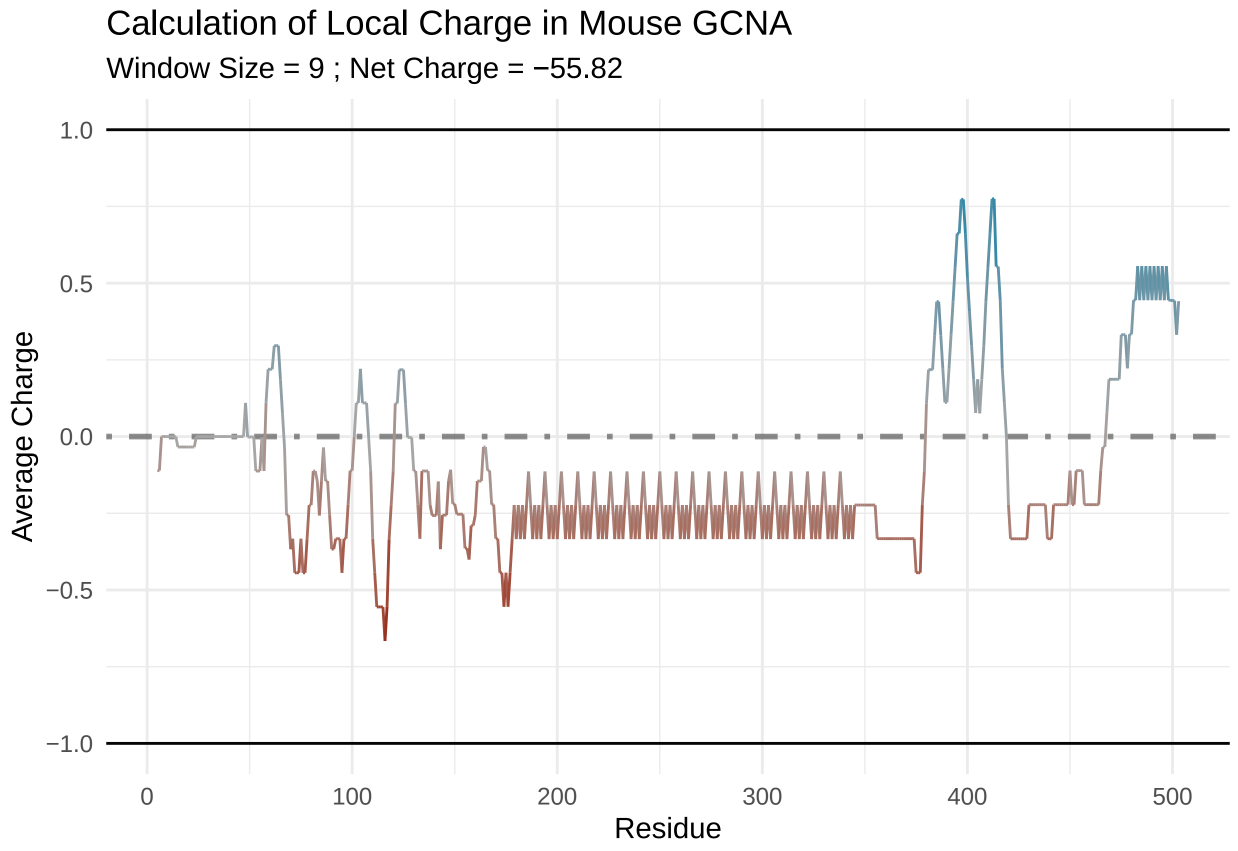
Calculation of Local Charge in Mouse GCNA
Window Size = 9 ; Net Charge = −55.82
1.0
0.5
0.0
−0.5
−1.0
Average Charge
0
100
200
300
400
500
Residue

```
##
## [[4]]
```

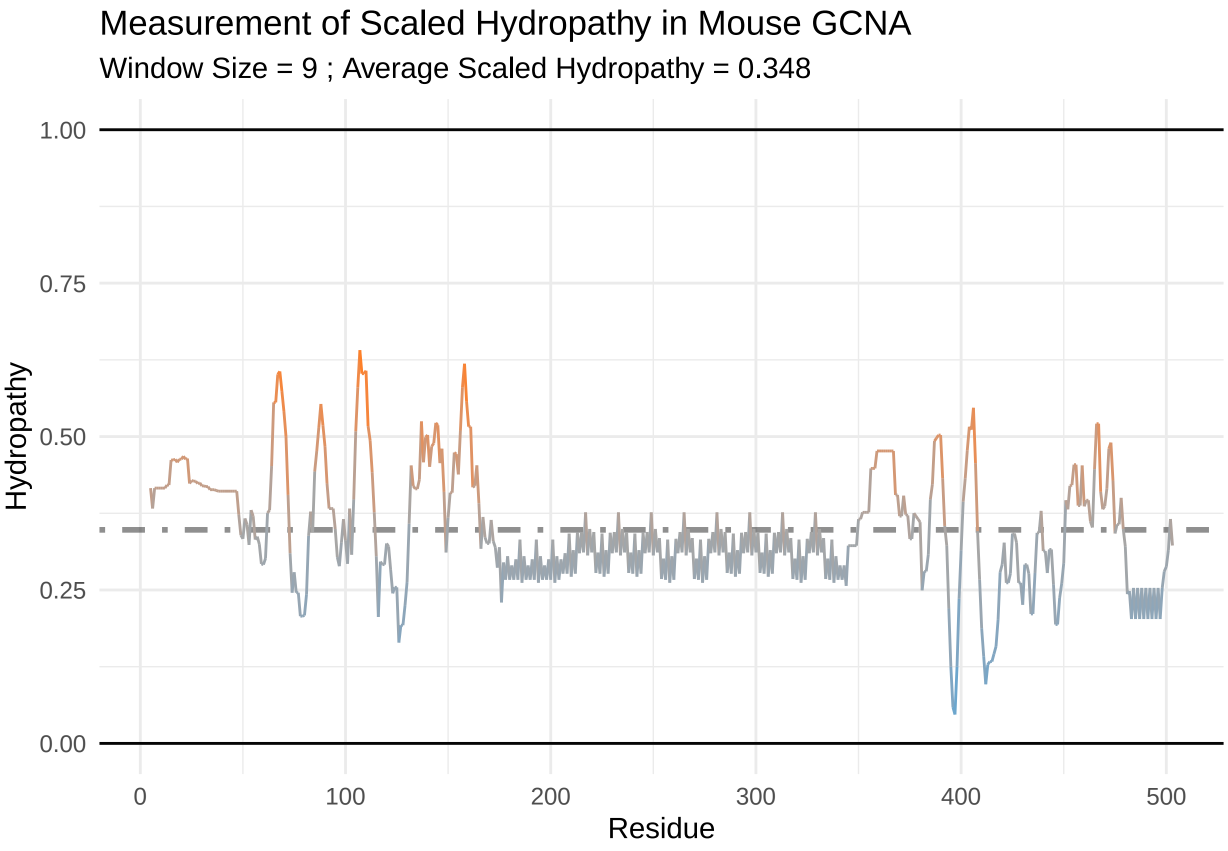

Measurement of Scaled Hydropathy in Mouse GCNA
Window Size = 9 ; Average Scaled Hydropathy = 0.348
Hydropathy
1.00
0.75
0.50
0.25
0.00
0
100
200
300
400
500
Residue


```
##
## [[5]]
```

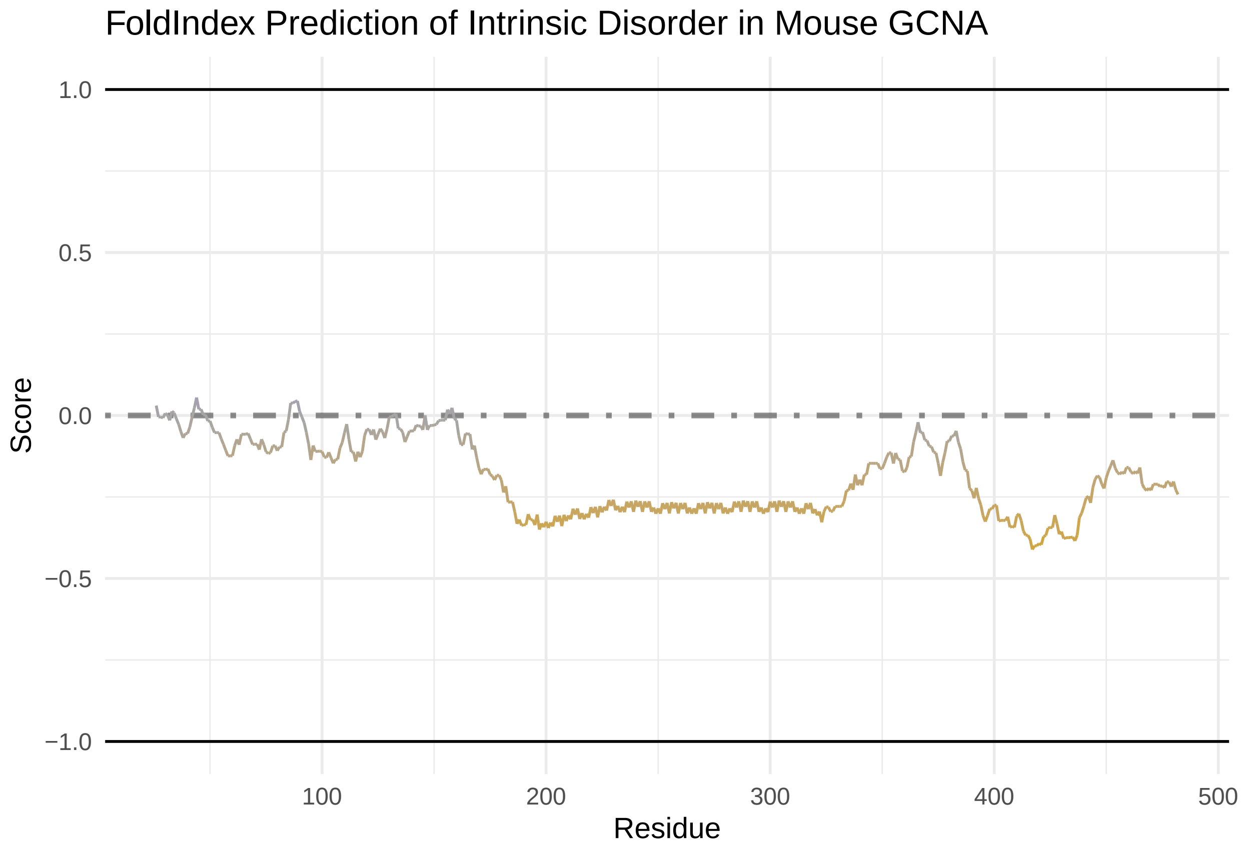
FoldIndex Prediction of Intrinsic Disorder in Mouse GCNA
1.0
0.5
0.0
−0.5
−1.0
Score
100
200
300
400
500
Residue

```
##
## [[6]]
```

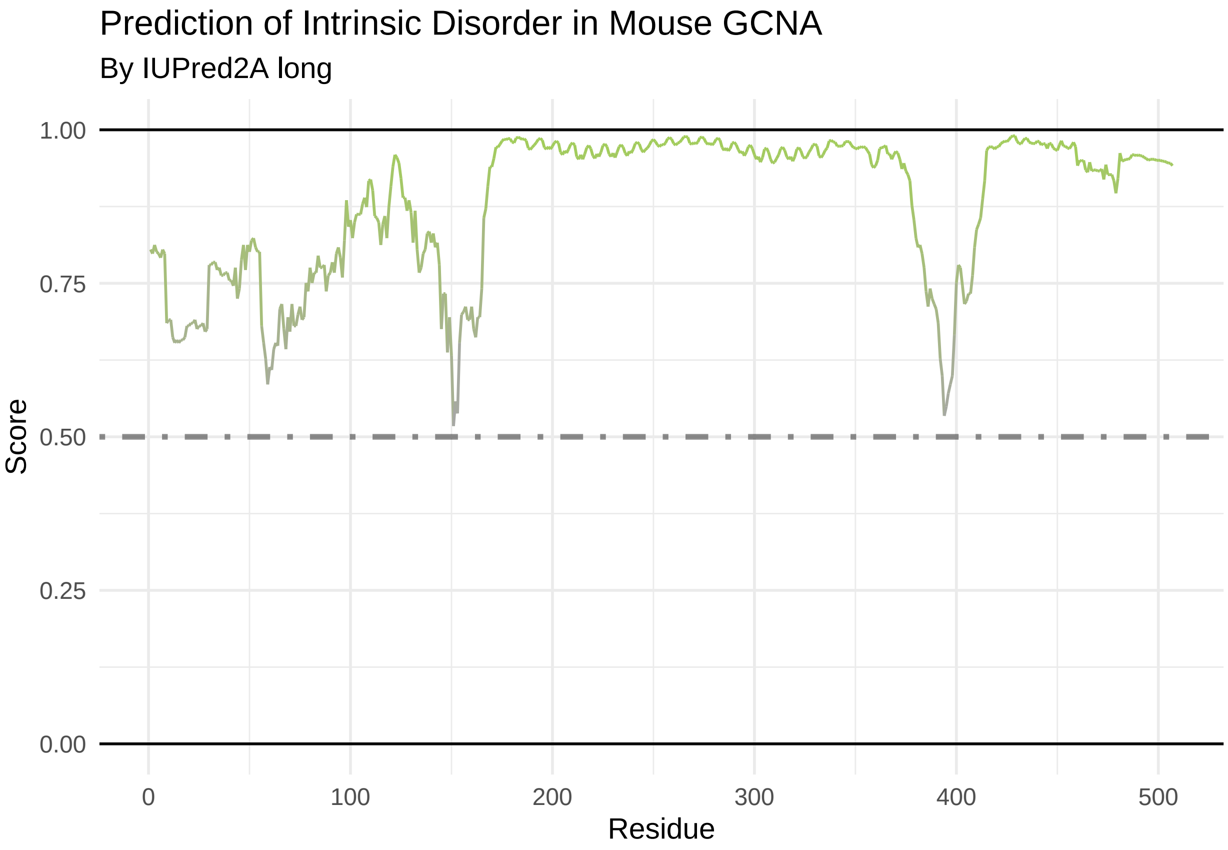

5 Appendix

5.1 References

5.1.1 Paper Citations

- Erdős, G., & Dosztányi, Z. (2020). Analyzing protein disorder with IUPred2A. Current Protocols in Bioinformatics, 70, e99. https://doi.org/10.1002/cpbi.99
- Mészáros, B., Erdős, G., & Dosztányi, Z. (2018). IUPred2A: context-dependent prediction of protein disorder as a function of redox state and protein binding. Nucleic acids research, 46(W1), W329-W337.
- UniProt Consortium. "UniProt: a hub for protein information." Nucleic acids research 43.D1 (2015): D204-D212.

5.1.2 R / Package Citations

```
citation()
```

```
##
## To cite R in publications use:
##
##   R Core Team (2022). R: A language and environment for statistical
##   computing. R Foundation for Statistical Computing, Vienna, Austria.
##   URL https://www.R-project.org/.
```

```
##
## A BibTeX entry for LaTeX users is
##
##   @Manual{,
##     title = {R: A Language and Environment for Statistical Computing},
##     author = {{R Core Team}},
##     organization = {R Foundation for Statistical Computing},
##     address = {Vienna, Austria},
##     year = {2022},
##     url = {https://www.R-project.org/},
##   }
##
## We have invested a lot of time and effort in creating R, please cite it
## when using it for data analysis. See also 'citation("pkgname")' for
## citing R packages.
```

```
citation("idpr")
```

```
##
## To cite package 'idpr' in publications use:
##
##   William M. McFadden and Judith L. Yanowitz (2020). idpr: Profiling
##   and Analyzing Intrinsically Disordered Proteins in R. R package
##   version 1.5.11.
##
## A BibTeX entry for LaTeX users is
##
##   @Manual{,
##     title = {idpr: Profiling and Analyzing Intrinsically Disordered Proteins in R},
##     author = {William M. McFadden and Judith L. Yanowitz},
##     year = {2020},
##     note = {R package version 1.5.11},
##   }
```

```
citation("Biostrings")
```

```
##
## To cite package 'Biostrings' in publications use:
##
##   H. Pagès, P. Aboyoun, R. Gentleman and S. DebRoy (2021). Biostrings:
##   Efficient manipulation of biological strings. R package version
##   2.62.0. https://bioconductor.org/packages/Biostrings
##
## A BibTeX entry for LaTeX users is
##
##   @Manual{,
##     title = {Biostrings: Efficient manipulation of biological strings},
##     author = {H. Pagès and P. Aboyoun and R. Gentleman and S. DebRoy},
##     year = {2021},
##     note = {R package version 2.62.0},
##     url = {https://bioconductor.org/packages/Biostrings},
##   }
##
```

```
## ATTENTION: This citation information has been auto-generated from the
## package DESCRIPTION file and may need manual editing, see
## 'help("citation")'.
```

```r
citation("ggplot2")
```

```
##
## To cite ggplot2 in publications, please use:
##
##   H. Wickham. ggplot2: Elegant Graphics for Data Analysis.
##   Springer-Verlag New York, 2016.
##
## A BibTeX entry for LaTeX users is
##
##   @Book{,
##     author = {Hadley Wickham},
##     title = {ggplot2: Elegant Graphics for Data Analysis},
##     publisher = {Springer-Verlag New York},
##     year = {2016},
##     isbn = {978-3-319-24277-4},
##     url = {https://ggplot2.tidyverse.org},
##   }
```

```r
citation("UniprotR")
```

```
##
## To cite UniprotR in publications use:
##
##   Soudy, M., Anwar, A.M., Ahmed, E.A., Osama, A., Ezzeldin, S.,
##   Mahgoub, S. and Magdeldin, S., 2020. UniprotR: Retrieving and
##   visualizing protein sequence and functional information from
##   Universal Protein Resource (UniProt knowledgebase). Journal of
##   Proteomics, 213, p.103613.
##
## A BibTeX entry for LaTeX users is
##
##   @Article{,
##     title = {UniprotR: Retrieving and visualizing protein sequence and functional information from Un
##     author = {Mohamed Soudy and Ali Mostafa Anwar and Eman Ali Ahmed and Aya Osama and Shahd Ezzeldin
##     journal = {Journal of Proteomics},
##     volume = {213},
##     pages = {103613},
##     year = {2020},
##     issn = {1874-3919},
##     doi = {10.1016/j.jprot.2019.103613},
##     url = {https://www.sciencedirect.com/science/article/pii/S1874391919303859},
##   }
```

5.2 Additional Information

5.2.1 Session Info

```
sessionInfo()
```

```
## R version 4.1.3 (2022-03-10)
## Platform: x86_64-apple-darwin17.0 (64-bit)
## Running under: macOS Big Sur/Monterey 10.16
##
## Matrix products: default
## BLAS:   /Library/Frameworks/R.framework/Versions/4.1/Resources/lib/libRblas.0.dylib
## LAPACK: /Library/Frameworks/R.framework/Versions/4.1/Resources/lib/libRlapack.dylib
##
## locale:
## [1] en_US.UTF-8/en_US.UTF-8/en_US.UTF-8/C/en_US.UTF-8/en_US.UTF-8
##
## attached base packages:
## [1] stats4    stats     graphics  grDevices utils     datasets  methods
## [8] base
##
## other attached packages:
## [1] Biostrings_2.62.0   GenomeInfoDb_1.30.1 XVector_0.34.0
## [4] IRanges_2.28.0      S4Vectors_0.32.3    BiocGenerics_0.40.0
## [7] ggplot2_3.3.5       idpr_1.5.11
##
## loaded via a namespace (and not attached):
##   [1] colorspace_2.0-3           seqinr_4.2-8
##   [3] ggsignif_0.6.3             ellipsis_0.3.2
##   [5] qdapRegex_0.7.2            GenomicRanges_1.44.0
##   [7] fs_1.5.2                   rstudioapi_0.13
##   [9] farver_2.1.0               ggpubr_0.4.0
##  [11] alakazam_1.2.0             fansi_1.0.3
##  [13] lubridate_1.8.0            xml2_1.3.3
##  [15] knitr_1.38                 ade4_1.7-18
##  [17] jsonlite_1.8.0             Rsamtools_2.8.0
##  [19] broom_0.7.12               dbplyr_2.1.1
##  [21] data.tree_1.0.0            readr_2.1.2
##  [23] compiler_4.1.3             httr_1.4.2
##  [25] backports_1.4.1            assertthat_0.2.1
##  [27] Matrix_1.4-1               fastmap_1.1.0
##  [29] lazyeval_0.2.2             cli_3.2.0
##  [31] htmltools_0.5.2            prettyunits_1.1.1
##  [33] tools_4.1.3                igraph_1.2.11
##  [35] gtable_0.3.0               glue_1.6.2
##  [37] GenomeInfoDbData_1.2.7     dplyr_1.0.8
##  [39] Rcpp_1.0.8.3               carData_3.0-5
##  [41] Biobase_2.52.0             cellranger_1.1.0
##  [43] vctrs_0.3.8                ape_5.6-2
##  [45] nlme_3.1-157               xfun_0.30
##  [47] stringr_1.4.0              networkD3_0.4
##  [49] rvest_1.0.2                lifecycle_1.0.1
##  [51] rstatix_0.7.0              zlibbioc_1.40.0
```

```
##  [53] MASS_7.3-56                 scales_1.1.1
##  [55] airr_1.3.0                  hms_1.1.1
##  [57] MatrixGenerics_1.4.3        parallel_4.1.3
##  [59] SummarizedExperiment_1.22.0 tidyverse_1.3.1
##  [61] gprofiler2_0.2.1            yaml_2.3.5
##  [63] curl_4.3.2                  gridExtra_2.3
##  [65] stringi_1.7.6               highr_0.9
##  [67] BiocParallel_1.26.2         rlang_1.0.2
##  [69] pkgconfig_2.0.3             matrixStats_0.61.0
##  [71] bitops_1.0-7                evaluate_0.15
##  [73] lattice_0.20-45             purrr_0.3.4
##  [75] UniprotR_2.1.0              labeling_0.4.2
##  [77] GenomicAlignments_1.28.0    htmlwidgets_1.5.4
##  [79] tidyselect_1.1.2            ggsci_2.9
##  [81] plyr_1.8.7                  magrittr_2.0.2
##  [83] R6_2.5.1                    magick_2.7.3
##  [85] generics_0.1.2              DelayedArray_0.18.0
##  [87] DBI_1.1.2                   pillar_1.7.0
##  [89] haven_2.4.3                 withr_2.5.0
##  [91] abind_1.4-5                 RCurl_1.98-1.6
##  [93] tibble_3.1.6                modelr_0.1.8
##  [95] crayon_1.5.1                car_3.0-12
##  [97] utf8_1.2.2                  plotly_4.10.0
##  [99] tzdb_0.2.0                  rmarkdown_2.13
## [101] progress_1.2.2              grid_4.1.3
## [103] readxl_1.3.1                data.table_1.14.2
## [105] forcats_0.5.1               reprex_2.0.1
## [107] digest_0.6.29               tidyr_1.2.0
## [109] munsell_0.5.0               viridisLite_0.4.0
```

5.2.2 Runtime

```
#--- End Runtime
end_time <- Sys.time()
time_diff <-  end_time - start_time
time_diff
```

```
## Time difference of 55.51599 secs
```
